# Standards for Methods Utilizing Environmental DNA for Detection of Fish Species

**DOI:** 10.3390/genes11030296

**Published:** 2020-03-11

**Authors:** Lu Shu, Arne Ludwig, Zuogang Peng

**Affiliations:** 1 Key Laboratory of Freshwater Fish Reproduction and Development (Ministry of Education), Southwest University School of Life Sciences, Chongqing 400715, China; shulu331@email.swu.edu.cn; 2 Department of Evolutionary Genetics, Leibniz-Institute for Zoo and Wildlife Research, 10315 Berlin, Germany; 3 Albrecht Daniel Thaer-Institute, Faculty of Life Sciences, Humboldt University Berlin, 10115 Berlin, Germany

**Keywords:** environmental DNA, water sampling, eDNA capture, eDNA extraction, eDNA detection, genetic marker, detection error

## Abstract

Environmental DNA (eDNA) techniques are gaining attention as cost-effective, non-invasive strategies for acquiring information on fish and other aquatic organisms from water samples. Currently, eDNA approaches are used to detect specific fish species and determine fish community diversity. Various protocols used with eDNA methods for aquatic organism detection have been reported in different eDNA studies, but there are no general recommendations for fish detection. Herein, we reviewed 168 papers to supplement and highlight the key criteria for each step of eDNA technology in fish detection and provide general suggestions for eliminating detection errors. Although there is no unified recommendation for the application of diverse eDNA in detecting fish species, in most cases, 1 or 2 L surface water collection and eDNA capture on 0.7-μm glass fiber filters followed by extraction with a DNeasy Blood and Tissue Kit or PowerWater DNA Isolation Kit are useful for obtaining high-quality eDNA. Subsequently, species-specific quantitative polymerase chain reaction (qPCR) assays based on mitochondrial cytochrome b gene markers or eDNA metabarcoding based on both *12S* and *16S rRNA* markers via high-throughput sequencing can effectively detect target DNA or estimate species richness. Furthermore, detection errors can be minimized by mitigating contamination, negative control, PCR replication, and using multiple genetic markers. Our aim is to provide a useful strategy for fish eDNA technology that can be applied by researchers, advisors, and managers.

## 1. Introduction

Environmental DNA (eDNA) is a newly developed and promising resource for species detection [1,2]. In contrast to DNA collected directly from organisms, eDNA is obtained from water, sediments, soil, or ice of various environmental samples [3,4,5] and reveals important information about past and present biodiversity [6]. eDNA from aquatic ecosystems is suitable for diversity assessment of contemporary aquatic species [7]. Furthermore, emerging eDNA technologies are non-invasive, sensitive, and cost-efficient compared to traditional survey approaches [2,5,8]. Target organisms can be detected at any life stage, including in the egg, larval, and juvenile forms from eDNA samples. Recently, eDNA was used to detect the critically endangered Mekong giant catfish *Pangasianodon gigas* [9] and rare green sturgeon *Acipenser medirostris*, demonstrating that the method is applicable for detecting rare fish species even in large river systems [10,11].

The promising applications of eDNA for monitoring aquatic biodiversity including fishes or their communities have been extensively reviewed [1,2,5,8,12,13,14,15,16,17,18,19,20,21,22,23]. With respect to eDNA, fish are currently the most studied group of macro-organisms in aquatic ecosystems [24]. Various eDNA protocols for detecting fish among different groups and habitats have been developed (see below). The process of developing eDNA techniques for aquatic organism detection has also been detailed in literature [1,19]. The first study of eDNA in fish revealed the presence of two invasive species of Asian carps in river ecosystems [25]. The methods used in the study were later developed to detect invasive or endangered species by conventional polymerase chain reaction (cPCR), quantitative PCR (qPCR), or digital droplet PCR (ddPCR) [26,27,28]. With advancements in high-throughput sequencing (HTS), additional applications have focused on assessing fish species richness through metabarcoding [29,30]. Consequently, the potential applications, cost-effectiveness, and evident simplicity of eDNA technology have made this approach extremely popular for fish detection.

Although Lear et al. [31], Kumar et al. [32], and Tsuji et al. [24] have summarized various protocols for each step in eDNA methods for aquatic organism detection and proposed standards or guides in the field, such criteria are not specific to fish detection and lack quantitative standards for general situations. Because fishes have been identified as the taxonomic group with greatest research interest in the context of eDNA, it is important to determine appropriate standards for fish eDNA surveys [33]. To supplement and publicize these standards, we describe the key criteria in this review.

**Figure 1:**
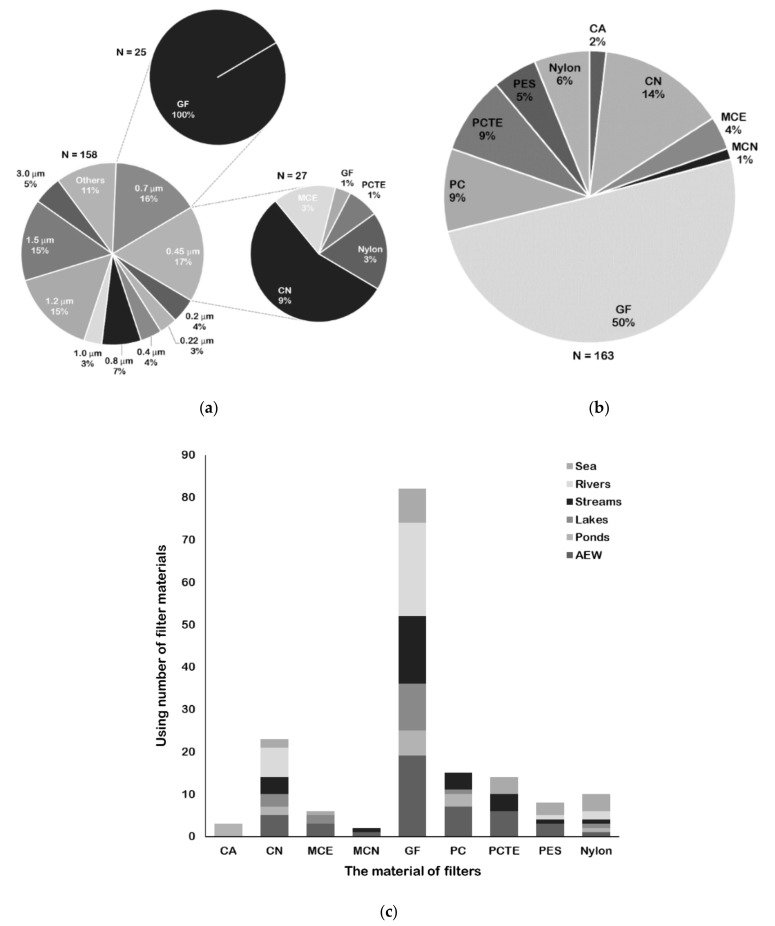
Various pore sizes and materials for filter types used for fish eDNA capture. (**a**) Percentage of pore size and two main pore sizes corresponding material of filters used in water filtration. (**b**) Percentage of various material of filters used in water filtration. (**c**) Using number of various material of filters in diverse water samples from different water environments. Abscissa shows various material of filter types, and ordinate represents corresponding using number. Different studied water samples form water environment types are displayed as different shades. AEW refer to artificial or experimental water bodies. The filter material abbreviations are cellulose acetate (CA), cellulose nitrate (CN), mixed cellulose acetate and nitrate (MCE), mixed cellulose nitrate (MCN), glass fiber (GF), polycarbonate (PC), polycarbonate track-etched (PCTE), and polyethersulfone (PES).

**Figure 2:**
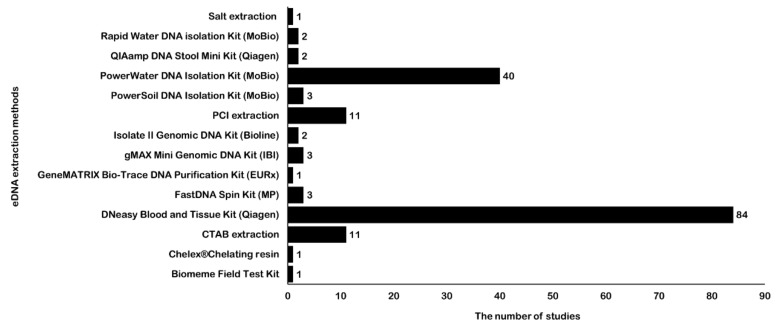
Application number of different eDNA extraction methods for fish detection. Abscissa shows the corresponding number of studies, and ordinate represents different eDNA extraction methods. The number of eDNA extraction methods is labeled following each bar. The abbreviations for extraction methods are cetyl trimethylammonium bromide (CTAB) and phenol-chloroform-isoamyl alcohol (PCI).

**Figure 3:**
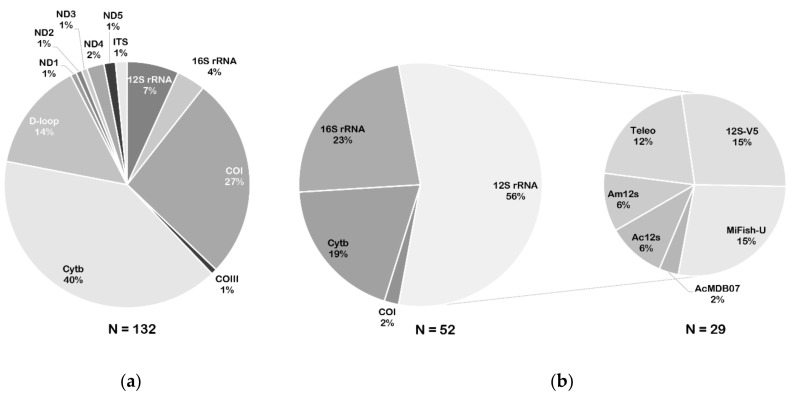
Percentages of genetic markers used for species-specific detection or eDNA metabarcoding. (**a**) Percentages of target genes used for species-specific polymerase chain reaction (PCR) assay. (**b**) Percentages of target genes and main marker corresponding primer sets used for metabarcoding analysis.

**Figure 4:**
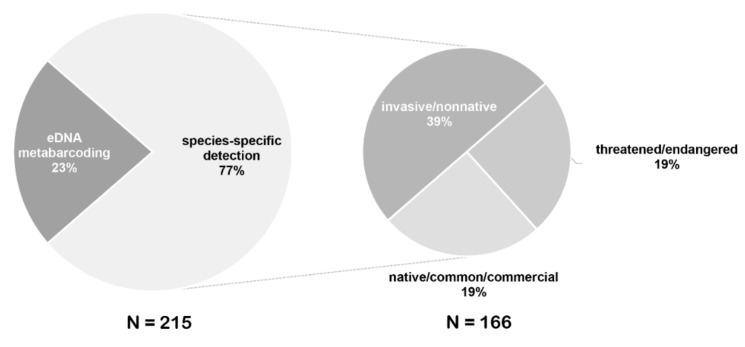
Percentages of eDNA studies employed for species-specific detection and eDNA metabarcoding. Percentages of different studied groups in species-specific detection are displayed in the smaller pie chart.

This review provides an overview of eDNA research including experimental and field studies of fishes performed for over a decade. We utilized “environmental DNA” and “fish” as keywords during a literature search on Web of Science. We manually filtered the papers using the criteria described by Tsuji et al. [24] and excluded review papers. The literature search was completed on 19th June 2019; a total of 168 research articles from 2011 to 2019 were included. We summarized detailed information from all papers (Table S1) and recorded technical protocols associated with each step of fish eDNA detection (Table S2). Based on this information, the frequency and proportion of use of each protocol were calculated from the 168 eDNA studies (Figure 1, Figure 2, Figure 3 and Figure 4).

## 2. Criteria for eDNA Sampling, Capture, and Extraction Strategies

### 2.1. Sample Volume, Depths, and Amount of Water

There are no general guidelines for sample volume, depths, and amounts of water. These factors must typically be considered according to the purpose of study, size and quality of the water body, potential abundance of target species, and subsequent steps of eDNA analysis. In fish eDNA detection, samples are collected from diverse water sources including artificial or experimental sources, ponds, lakes, streams, rivers, and seas and are obtained from different depths including the surface, middle, bottom, or others (Table S2). Sample volumes vary from 1.5 mL [34] to 45 L [35]. However, 1 or 2 L is typically collected from the field (Table S2). The standard volume of 1 or 2 L of water is most commonly utilized for filtration and concentrating DNA from water samples (Table S2). An appropriate sampling depth improves species detection, which should differ between lotic (e.g., stream) and lentic (e.g., lake) ecosystems. For example, streams tend to have a turbulent flow with well-mixed water; thus, surface water samples can be used for eDNA detection in streams [36]. Lake stratification can lead to differences in the depth at which a sample is collected; thus, pooling different layers of water for each sample containing at least upper and lower water is recommended to reduce sampling bias for diversity assessment in lentic systems [37]. The sample volume depends on the size of the water body and abundance of the target species. If the surveyed water body is a large lake (e.g., a lake with a surface area of 535 ha [38]) or coastal sea [39], more samples must be collected to include more eDNA molecules. If the target species is widely distributed or has low abundance, the sampling range and volume should also be expanded to capture as many of the target species as possible [40]. Information about the ecology (e.g., feeding areas or spawning grounds) of target species should be used for sampling. Field sample replicates are necessary to enhance the efficiency of DNA collection and probability of target eDNA detection; at least three samples were collected from each site in most studies [38,41,42,43,44,45]. When the number of samples is increased, to improve work efficiency and reduce analysis cost, researchers may consider pooling the water samples from multiple locations for subsequent processing. However, Sato et al. [46] and Zhang et al. [47] demonstrated that pooled samples decreased species detection compared to using individually processed samples, indicating that sample pooling is not useful for assessing fish assemblages. Thus, we do not recommend pooling samples for subsequent analysis. In addition, for lentic systems such as lakes, shoreline sampling with surface water can capture most fish species eDNA [47]. We recommend that shoreline sampling can be used to briefly survey lentic systems, whereas systematic spatial sampling should be used in subtle spatial distribution pattern surveys for entire fish species in large lentic systems (e.g., a lake with a surface area of 4343 ha [47]).

### 2.2. Methods of eDNA Concentration

The methods of eDNA concentration involve centrifugation, isopropanol or ethanol precipitation, and filtration. Centrifugation and precipitation are generally suitable for collecting small volumes such as 1.5 mL [34] or 15 mL [48] of water, whereas filtration can handle larger bulk volumes from 250 mL to 45 L of water (Table S2). Filtration of a large volume of water can improve the detection of rare species, and preservation of the filters is more convenient than using water samples; hence, filtration methods have become the most widely used methods for fish eDNA capture from water samples. Filtering on-site is beneficial for eDNA preservation because it prevents eDNA decay during transportation, whereas filtering in a laboratory can save field time and eliminate field contamination. Based on the specific survey water and field conditions, one filtration strategy can be chosen. When the water has low turbidity, number of samples is small, and portable pump has enough power to process samples, we recommend on-site filtration [36,49]. When sampling turbid water or the sample number is large, water samples should be transported back to the laboratory in a sterile bottle for filtration [50,51]. Additionally, we recommend water sample transport under dark conditions because ultraviolet (UV) light is damaging to DNA. Because of the difficultly in achieving refrigeration or freezing in the field, we recommend using a liquid preservative (e.g., ethanol [52]) to store eDNA on filters or water samples at room temperature. Most studies suggest that the sampling to filtering steps should be conducted within 24 h of collection (Table S2). At a remote survey site, water samples should be filtered locally rather than transported back to the main laboratory, which also reduces transportation costs [53]. Furthermore, enclosed capsule filters (e.g., Sterivex^TM^-GP unit [54]), which are not opened in the field or in some cases not even in the laboratory, is a good choice for the collection and transportation of field water samples, reducing contamination throughout eDNA sampling.

### 2.3. Selection of Filters for Fish eDNA Capture

Various pore sizes and materials for filter types have been reported (Table S2). The reported pore sizes of filters vary from 0.2–180 μm, and the most commonly used pore size is 0.45 μm (Table S2; Figure 1a). It was reported that 1–10 μm particles are the most common sizes of fish DNA molecules obtained from water [55,56,57]. Thus, selecting a corresponding range of pore sizes may ensure the success of fish eDNA capture. The eDNA of water samples can be effectively collected with cellulose acetate (CA), cellulose nitrate (CN), mixed cellulose acetate and nitrate (MCE), mixed cellulose nitrate (MCN), glass fiber (GF), polycarbonate (PC), polycarbonate track-etched (PCTE), polyethersulfone (PES), and nylon filters (Table S2). The percentage of filter materials used in fish eDNA collection is shown in Figure 1b. GF filers are most common and are used in 50% of cases, followed by CN filters (14%). Although Kumar et al. [32] concluded that cellulose-based filters consistently performed better than other filters based on the results of eDNA studies for aquatic organisms, there is some controversy regarding whether cellulose-based filters or GF filters are the most suitable filter types for fish DNA capture. In experimental aquariums, Renshaw et al. [58] and Hinlo et al. [52] found that using CN filters yielded higher eDNA copies than using other filters for bluegill sunfish *Lepomis macrochirus* and oriental weather loach *Misgurnus anguillicaudatus*, respectively. In a field survey, Sepulveda et al. [59] found that a 1.0 μm MCE filter resulted in higher detection rates than a 1.2 μm GF filter for northern pike *Esox lucius* in lakes. However, in experimental aquariums, Eichmiller et al. [48] and Minamoto et al. [60] recommended that GF filters are optimal for collecting eDNA from common carp *Cyprinus carpio*. Lacoursiere-Roussel et al. [61] reported that GF filters performed better than MCE filters for brook charr *Salvelinus fontinalis* detection. Because of the different water bodies and target species in these studies, a general criterion cannot be drawn for future reference. Although it is recommended to conduct a pre-experiment to determine a suitable capture method before performing a formal survey, it is also important to give a general choice in advance. Here, we recommend using GF filters for fish eDNA capture, as they have been commonly used in diverse water samples from aquarium water, lentic systems, or lotic systems for fish detection (Figure 1c). Additionally, 0.7 μm is a general pore size of GF filters used for water filtration (Figure 1a); however, when filtering turbid water samples, a larger pore size (>1 μm) should be considered to avoid filter logging. If researchers wish to simultaneously use more than one type of filter, capsule filters may be required which can also contain two filter membranes of different pore sizes and materials [62].

### 2.4. eDNA Extraction Methods

For fish eDNA detection, in addition to the few studies that used cetyl trimethylammonium bromide (CTAB), phenol-chloroform-isoamyl alcohol (PCI), or salt DNA extraction methods, most studies have employed different commercial DNA extraction kits for eDNA extraction (Table S2, Figure 2). The most widely used eDNA extraction method is the DNeasy Blood and Tissue Kit (Qiagen, Hilden, Germany), followed by the PowerWater DNA Isolation Kit (MoBio, Hilden, Germany). Kumar et al. [32] and Tsuji et al. [24] compared the advantages and disadvantages of different methods for eDNA extraction. They found that the DNeasy Blood and Tissue Kit was optimal for eDNA extraction in most cases because it is non-toxic, simple, and less costly than other kits. The cost of PowerWater kit is higher than the DNeasy kit, but its PCR inhibitor removal can effectively improve PCR amplification and data quality [15,63]. Stoeckle et al. [64] systematically evaluated the influence of different environmental variables and inhibitors and found that the presence of sediment was the main factor responsible for lower eDNA detection in the water samples, regardless of whether flowing or still water was used. Determining such information beforehand can help decide whether a protocol involving inhibitor removal is required. Here, we recommend using the PowerWater DNA Isolation Kit for water samples containing humic substance, algae, or siliceous of sediment particles because of its PCR inhibitor removal step.

## 3. Genetic Marker Selection

Appropriate genetic markers and primers vary depending on the purpose of eDNA detection (Table S2). Specific primers are designed for single species detection, whereas generic primers are designed for diverse taxa assessment through metabarcoding. Mitochondrial and nuclear genes have already been utilized as genetic markers in eDNA assays; however, mitochondrial genes are considered as gold standard because they rapidly evolve and can better describe biodiversity; moreover, mitochondrial DNA has been shown to be reliable for evaluating degraded DNA, is informative for discriminating vertebrate species, and is adapted to survey fish diversity [5,65]. The commonly used markers for species-specific detection through fish eDNA are the cytochrome b gene (*Cytb*; 70–150 bp), cytochrome oxidase subunit I gene (*COI*; 61–240 bp), and *D-loop* region (98–312 bp) (Table S3; Figure 3a). As *Cytb* is the most popular genetic marker for characterizing eDNA from fish (Figure 3a), we suggest that investigators give priority to designing species-specific primers based on *Cytb* for target species detection. In this review, 18 pairs of universal primers for fish eDNA metabarcoding were evaluated (Table S3). The *12S* rRNA markers have been utilized extensively in fish diversity assessments (Figure 3b). Based on the results of *12S* rRNA, the 12S-V5 [66], MiFish-U [29], and Teleo [30] primer sets are recommended for fish diversity analysis, as they provide the highest percentages of all *12S* rRNA markers used (15%, 15%, and 12%, respectively; Figure 3b). Bylemans et al. [67] developed multiple software programs to evaluate the performance of 14 sets of universal primers in richness assessment of freshwater fish species from the Murray-Darling (Australia). The results showed that the MiFish-U, Teleo, and AcMDB07 primers were useful for fish eDNA metabarcoding surveys; however, the MiFish-U and AcMDB07 primers provided higher species detection probabilities compared to the Teleo primer set. Thus, before confirming the most suitable primer set, it is imperative to carefully estimate the history of local biodiversity and formulate the objectives for a metabarcoding survey. In addition, *16S* rRNA is the second most popular genetic marker examined in fish diversity analysis. Here, combined use of the 12S and 16S markers is recommended to increase eDNA metabarcoding detection of target fish communities and reduce metabarcoding detection bias (see below).

## 4. eDNA Detection

eDNA detection methods used in fish monitoring mainly include two types of species-specific detection and eDNA metabarcoding, which were described in detail in several reviews [1,5,19,24,32]. Most eDNA studies of fish monitoring have focused on species-specific detection including the detection of invasive and threatened species (Figure 4). Early detection of invasive species or evaluation of their eradication is essential for developing conservation strategies to protect the diversity of native species. Because of its sensitivity and cost-effectiveness, eDNA technology has recently become increasingly used as a management strategy for monitoring invasive fish before and after their eradication, such as Asian carps in the Great Lakes of the United States and Canada [25,40,41,68,69,70]. eDNA analysis can screen the presence or absence of rare fish species in water samples; thus, it has become very useful for monitoring and conserving vulnerable species. eDNA sampling has also become a common tool for surveying at-risk species because of its non-invasive nature and cost-effectiveness, such as for evaluating Green sturgeon [10,11] in the United States, Chinese sturgeon [71], and Mekong giant catfish [9] in Asia. In addition to the absence-presence detection of single species, estimating the species richness of fish communities is important for management and conservation. eDNA metabarcoding through HTS is a promising and powerful tool for fish diversity surveys.

Initial eDNA detection employs cPCR with cloning or Sanger sequencing technologies to obtain target eDNA for species-specific detection [25,40,72,73]. Here, we recommend probe-based qPCR for target eDNA detection, which is preferred over cPCR in single-species detection because of its greater sensitivity and reliability [74,75,76]. Moreover, qPCR methods, which can quantify the target eDNA in samples, have been used to estimate fish abundance and biomass [77,78]. Methods involving ddPCR are also used in target species detection or quantification, as they are more sensitive at low eDNA concentrations. Doi et al. [28,79] found that ddPCR was more effective than qPCR for eDNA detection or quantification of rare invasive species, such as bluegill sunfish and common carp. However, the assay costs of ddPCR are higher than those of qPCR [80]. Thus, before cost reduction of the ddPCR assay, we do not recommend performing ddPCR for single-species detection.

In addition to the lab-based eDNA approaches described above, a portable field-based eDNA platform with a field-based DNA extraction kit, shelf-stable assay, and portable real-time PCR thermocycler have been recently employed in a species-specific eDNA survey. Sepulveda et al. [59] compared the performance of a portable field-based eDNA platform to lab-based eDNA approaches for detecting invasive northern pike in eight lakes on Alaska’s Kenai Peninsula. The portable, field-based platform is less time-consuming (~1 h) than lab-based approaches from water collection to final results, but is less sensitive than lab-based approaches and more prone to inhibition, thus increasing the potential of obtaining false-negative results. Until these sensitivity and inhibition issues can be resolved, this portable field-based approach is not recommended as a substitute for lab-based eDNA approaches.

HTS can be advantageous when targeting multiple species via eDNA metabarcoding, making it possible to eliminate the costly and time-consuming steps of cloning and sequencing of single PCR products by Sanger sequencing [3]. A detailed comparison of many sequencing platforms and approaches used for eDNA metabarcoding analysis was conducted by Lear et al. [31]. The authors concluded that the low cost per sequence makes Illumina platforms suitable for high-throughput analysis of eDNA metabarcoding, allowing for high throughput and large coverage. Lear et al. [31] also mentioned that the emerging portable sequencing technology allowing real-time sequencing in the field has great potential for eDNA research; however, its error rates must be further improved. Furthermore, Deiner et al. [81] amplified and sequenced longer fragments separately from fish eDNA from a mesocosm and natural stream and successfully recovered the mitogenomes of several species from fish eDNA in vitro, in situ, and in the field, indicating that not all eDNA is degraded. In the future, sequencing of mitochondrial genomes from eDNA samples may overcome the low resolution and species identification observed with short-fragment PCR amplicon-based methods.

## 5. Detection Errors

eDNA technology remains challenging when detecting errors such as false-positives and false-negatives. False-positives refer to the apparent detection of a species despite its absence in the environment, whereas false-negatives in eDNA analysis refer to the failure to detect the DNA of a species despite its presence in the environment [82,83]. Either false-positives or false-negatives make eDNA results uncertain in terms of the absence or presence of a species. Methodological errors and environmental factors are the two major factors that can cause false detections. Understanding the origin, state, transport, and fate of eDNA in aquatic ecosystems [6,84,85] can help explain uncertainties in eDNA detection; additionally, standards and guidelines must also be developed to ensure the adequacy of quality assurance, as was performed in ancient DNA research [86]. Furlan et al. [87,88] developed a framework for reducing or eliminating potential false-positive or false-negative detection during eDNA analysis. Thomsen et al. [1] and Cristescu et al. [89] also provide detailed recommendations for field sampling designs and lab practices to reduce or avoid false detections in eDNA studies. Detailed solutions to these issues can be found in the literature cited above. Here, we highlight some key standards in field sampling and laboratory levels to eliminate methodological errors.

### 5.1. Contamination Control

Exogenous or non-target contaminating DNA is ubiquitous during eDNA processing, which is the dominant factor causing false-positives [90]. Based on the strict decontamination protocols of ancient DNA research [6,91], spatial separation and surface cleaning are effective methods for avoiding sample, lab, or cross-contamination. Thus, we recommend that eDNA analysis should be done in separated laboratories (i.e. sample preparation room, DNA extraction room and amplification/sequencing room) which are used in a one-way system by qualified researchers only. Researchers should wear suitable lab clothes for trace labs. All equipment must be bleached with 10% bleach solution or treated with 254 nm ultraviolet light prior to sampling, filtering, and pre-PCR steps. When filtering water samples, the contact surface of the filters for each sample should also be treated with 10% bleach and rinsed with double-distilled H_2_O before new sample filtration [48,58] to prevent cross-contamination between samples. Furthermore, strictly setting up multiple negative controls (such as double-distilled H_2_O for blank sample) including a field blank, filtration blank, extraction blank, and PCR blank [47,92], is necessary for evaluating contamination during the entire eDNA process. In addition, considering the repeatability of eDNA results, analysis of at least some samples should be independently repeated in another laboratory as described for ancient DNA research [6,91].

### 5.2. Reducing Primer Bias

Primer bias occurs in PCR amplification of eDNA templates during metabarcoding, such as mismatch between primers and templates or unequal amplification efficiency, resulting in false-negative detection of the target taxon or low-abundance species [15]. PCR replicates or the use of multiple genetic markers in PCR amplification increases the chance of amplifying low levels of eDNA from rare species or overcome a shortage of single genetic markers, which can improve target detection and reduce primer bias [83,93]. We recommend performing triplicate PCRs for each sample and pooling the samples to minimize bias in individual PCRs [38,94]. To increase eDNA metabarcoding detection of target fish communities, we recommend using both the 12S and 16S markers (Table S3), which is a common solution employed to eliminate PCR amplification bias in previous studies [95,96,97,98].

## 6. Conclusions

Although species detection using eDNA has many advantages, this method cannot fully substitute classical monitoring, particularly if information on population demography is needed for conservation management (for advantages and disadvantages, see Stoeckle et al. [99]). Despite its technical challenges, eDNA remains a promising and powerful tool for fish monitoring and conservation. In the last decade, eDNA methods have been increasingly utilized in fish detection for monitoring and conserving fish diversity. Numerous standards have been published in international journals; however, few investigators follow these standards in the field. Various protocols for eDNA methods prevent beginners from efficiently performing eDNA research, and published results are difficult with respect to their reliability. Here, we supplement and emphasize important standards of eDNA technology, particularly water collection, eDNA capture and extraction, genetic marker selection, and eDNA detection in fish surveys. We also highlighted key standards for reducing or avoiding false and negative detection in eDNA studies. Overall, our literature survey suggests that in most cases of fish detection, 1 or 2 L surface water collection, and eDNA capture on 0.7 μm GF filters, followed by extraction with the DNeasy Blood and Tissue Kit or PowerWater DNA Isolation Kit can be performed to obtain high-quality eDNA and reliable results. Subsequently, using specific primers based on *Cytb* for species-specific qPCR assay or using universal primers based on both *12S* rRNA and *16S* rRNA for eDNA metabarcoding via HTS is effective for target species identification or species richness assessment. We can also establish quality controls to minimize detection errors by mitigating contamination including spatial separation, surface cleaning, and negative control in each step of eDNA analysis, performing PCR replication such as triplicate PCRs for each sample, and using both 12S and 16S markers in multiple primer sets. Decontamination and negative control are aimed at decreasing false-positives, whereas PCR replication and using multiple genetic markers are aimed at decreasing false-negatives. Nevertheless, these standards must be adapted occasionally considering technical progress. The emergence of the portable field-based eDNA platform and portable sequencing technology suggest that with additional development and improvements, eDNA techniques can be used successfully to rapidly evaluate environmental samples.

## Supplementary Materials

The following are available online at https://www.mdpi.com/2073-4425/11/3/296/s1: Table S1: Fish eDNA studies from 2011 to 2019 reviewed in this paper; Table S2: Summary of sampling volume and depth, eDNA extraction, genetic markers, amplification and sequencing used to detect fish eDNA in water bodies; Table S3: 18 pairs of universal primers used in eDNA metabarcoding for fish diversity assessments.
