# Supplementary material for: Standards for Methods Utilizing Environmental DNA for Detection of Fish Species
Source: Genes (Basel). 2020 Mar 11;11(3):296. doi: 10.3390/genes11030296 (PMC7140814; doi:10.3390/genes11030296)
Supplement: Supplementary file 1 [file genes-11-00296-s001.zip › genes-712794-supplementary/Supplementary/Table S1.docx]

| Table S1. Fish eDNA studies from 2011 to 2019 reviewed in this paper. | | | | | | |
| --- | --- | --- | --- | --- | --- | --- |
| Reference | Title | Environment | Species | Groups studied | Geographical  location | Main research question |
| Jerde et al. (2011) | “Sight-unseen” detection of rare aquatic species using environmental DNA | rivers | Bighead and Silver Carps | invasive | Chicago, USA | invasive species detection |
| Dejean et al. (2011) | Persistence of environmental DNA in freshwater ecosystems | artificial ponds | Siberian Sturgeon | common | France | eDNA persistence |
| Minamoto et al. (2011) | Surveillance of fish species composition using environmental DNA | experimental aquariums; natural river | Freshwater fish | fish community | Japan | species composition |
| Takahara et al. (2012) | Estimation of fish biomass using environmental DNA | experimental aquariums and ponds; natural lagoon | Common Carp | common | Japan | biomass estimation |
| Thomsen et al. (2012a) | Monitoring endangered freshwater biodiversity using environmental DNA | ponds and streams | European Weather Loach | endangered | Germany and Poland | endangered species detection |
| Thomsen et al. (2012b) | Detection of a diverse marine fish fauna using environmental DNA from seawater samples | sea | Marine fish (15 species) | fish community | Denmark | species composition |
| Jerde et al. (2013) | Detection of Asian carp DNA as part of a Great Lakes basin-wide surveillance program | the Great Lakes basin (streams, rivers and bays) | Bighead and Silver Carps | invasive | Illinois, USA | invasive species detection |
| Takahara et al. (2013) | Using environmental DNA to estimate the distribution of an invasive fish species in ponds | ponds | Bluegill Sunfish | invasive | Japan | invasive species detection |
| Wilcox et al. (2013) | Robust detection of rare species using environmental DNA: the importance of primer specificity | streams | Brook Trout; Bull Trout | invasive; threatened | Montana, USA | invasive and threatened species detection |
| Barnes et al. (2014) | Environmental conditions influence eDNA persistence in aquatic systems | experimental mesocosms | Common Carp | invasive | Indiana, USA | eDNA persistence: degradation |
| Diaz-Ferguson et al. (2014) | Development of molecular markers for eDNA detection of the invasive African jewelfish (*Hemichromis letourneuxi*): a new tool for monitoring aquatic invasive species in National Wildlife Refuges | experimental aquariums; natural river | African Jewelfish | invasive | Loxahatchee, USA | invasive species detection and density estimation |
| Kelly et al. (2014) | Using environmental DNA to census marine fishes in a large mesocosm | artificial aquarium | Marine fish (8 species) | fish community | California, USA | fish diversity assessment and quantification via eDNA metabarcoding |
| Keskin (2014) | Detection of invasive freshwater fish species using environmental DNA survey | rivers | Prussian Carp, North African Catfish, Nile Tilapia and Topmouth Gudgeon | invasive | Turkish | invasive species detection |
| MacDonald et al. (2014) | Primers for detection of Macquarie perch from environmental and trace DNA samples | river | Macquarie Perch | endangered | Australia | endangered species detection |
| Mahon et al. (2014) | Meta-genomic surveillance of invasive species in the bait trade | artificial tanks | Freshwater fish | invasive | the Great Lakes, USA | non-target invasive species detection via eDNA metabarcoding |
| Turner et al. (2014) | Particle size distribution and optimal capture of aqueous macrobial eDNA | pond and lake | Common Carp | invasive | Indiana, USA | eDNA persistence: particle size |
| Wilson et al. (2014) | Tracking ghosts: combined electrofishing and environmental DNA surveillance efforts for Asian carps in Ontario waters of Lake Erie | lakes and rivers | Bighead, Silver and Grass Carps | invasive | Canada | invasive species detection |
| Wittmann et al. (2014) | Grass carp in the Great Lakes region: establishment potential, expert perceptions, and re-evaluation of experimental evidence of ecological impact | the Great Lakes basin (rivers and lakes) | Grass Carp | invasive | the Great Lakes, USA | invasive species detection |
| Amberg et al. (2015) | Improving efficiency and reliability of environmental DNA analysis for silver carp | the Great Lakes basin (rivers and lakes) | Silver Carp | invasive | the Great Lakes, USA | invasive species detection |
| Doi et al. (2015) | Droplet Digital Polymerase Chain Reaction (PCR) outperforms Real-Time PCR in the detection of environmental DNA from an invasive fish species | ponds | Bluegill Sunfish | invasive | Japan | invasive species detection |
| Doi et al. (2015) | Use of Droplet Digital PCR for estimation of fish abundance and biomass in environmental DNA Surveys | experimental mesocosms | Common Carp | common | Japan | abundance and biomass estimation |
| Evans et al. (2015) | Quantification of mesocosm fish and amphibian species diversity via environmental DNA metabarcoding | experimental mesocosms | Freshwater fish (8 species) and amphibian (1 species) | fish community | Indiana, USA | fish diversity assessment and quantification via eDNA metabarcoding |
| Furlan et al. (2015) | A framework for estimating the sensitivity of eDNA surveys | streams | Oriental Weather Loach | common | Australia | method optimization: estimating concentration and sensitivity of eDNA method |
| Jane et al. (2015) | Distance, flow and PCR inhibition: eDNA dynamics in two headwater streams | streams | Brook Trout | invasive | Massachusetts, USA | eDNA transport |
| Janosik et al. (2015) | Environmental DNA as an effective tool for detection of imperiled fishes | streams | Slackwater Darter | threatened | Tennessee, USA | threatened species detection |
| Klymus et al. (2015) | Quantification of eDNA shedding rates from invasive bighead carp *Hypophthalmichthys nobilis* and silver carp *Hypophthalmichthys molitrix* | experimental aquariums | Bighead and Silver Carps | invasive | Missouri, USA | eDNA production: eDNA shedding rates quantification |
| Laramie et al. (2015) | Characterizing the distribution of an endangered salmonid using environmental DNA analysis | rivers | Chinook Salmon | endangered | Washington, USA | endangered species detection |
| Miya et al. (2015) | MiFish, a set of universal PCR primers for metabarcoding environmental DNA from fishes: detection of more than 230 subtropical marine species | experimental aquariums; sea | Marine fish (230 species) | fish community | Okinawa, Japan | fish diversity assessment via eDNA metabarcoding |
| Nathan et al. (2015) | The use of environmental DNA in invasive species surveillance of the Great Lakes commercial bait trade | artificial tanks | Bighead and Silver Carps, Goldfish, Eurasian Rudd, Tubenose Goby and Round Goby | invasive | the Great Lakes, USA | invasive species detection |
| Renshaw et al. (2015) | The room temperature preservation of filtered environmental DNA samples and assimilation into a phenol-chloroform-isoamyl alcohol DNA extraction | experimental mesocosms | Bluegill Sunfish | invasive | USA | method optimization of eDNA capture, preservation, extraction |
| Schultz et al. (2015) | Modeling the Sensitivity of Field Surveys for Detection of Environmental DNA (eDNA) | rivers | Bighead and Silver Carps | invasive | the Great Lakes, USA | modeling the sensitivity of eDNA detection |
| Sigsgaard et al. (2015) | Monitoring the near-extinct European weather loach in Denmark based on environmental DNA from water samples | ponds and streams | European Weather Loach | endangered | Denmark | threatened species detection |
| Takahara et al. (2015) | Effects of sample processing on the detection rate of environmental DNA from the Common Carp (*Cyprinus carpio*) | ponds | Common Carp | common | Japan | method optimization: effects of sample processing on eDNA detection rate |
| Turner et al. (2015) | Fish environmental DNA is more concentrated in aquatic sediments than surface water | experimental ponds; natural rivers | Bighead Carp | invasive | Missouri, Indiana and Kansas, USA | method optimization of eDNA sampling |
| Wilcox et al. (2015) | Environmental DNA particle size distribution from Brook Trout (*Salvelinus fontinalis*) | stream | Brook Trout | invasive | Montana, USA | eDNA persistence: particle size |
| Adrian-Kalchhauser et al. (2016) | An eDNA assay to monitor a globally invasive fish species from flowing freshwater | ponds and rivers | Ponto-Caspian Gobies (5 species) | invasive | Germany | invasive species detection |
| Baldigo et al. (2016) | Efficacy of environmental DNA to detect and quantify Brook Trout populations in headwater streams of the Adirondack Mountains, New York | streams | Brook Trout | invasive | New York, USA | invasive species detection and abundance estimation |
| Banks et al. (2016) | Monitoring brown trout (*Salmo trutta*) eradication in a wildlife sanctuary using environmental DNA | streams | Brown Trout | invasive | New Zealand | invasive species detection |
| Bergman et al. (2016) | Detection of adult Green Sturgeon using environmental DNA analysis | river | Green Sturgeon | threatened | California, USA | threatened species detection |
| Boothroyd et al. (2016) | Environmental DNA (eDNA) detection and habitat occupancy of threatened spotted gar (*Lepisosteus oculatus*) | lakes | Spotted Gar | threatened | Southern Ontario, Canada | threatened species detection and abundance estimation |
| Bylemans et al. (2016) | Improving the containment of a freshwater invader using environmental DNA (eDNA) based monitoring | streams | Redfin Perch | invasive | Australia | invasive species detection |
| Cannon et al. (2016) | In silico assessment of primers for eDNA studies using PrimerTree and application to characterize the biodiversity surrounding the Cuyahoga River | river | Freshwater fish | fish community | Northeast Ohio, USA | fish diversity assessment via eDNA metabarcoding |
| Carim et al. (2016a) | An environmental DNA assay for detecting Arctic grayling in the upper Missouri River basin, North America | streams and rivers | Arctic Grayling | invasive | Missouri, USA | invasive species detection |
| Carim et al. (2016b) | An environmental DNA marker for detecting nonnative brown trout (*Salmo trutta*) | streams | Brown Trout | invasive | Missoula, USA | invasive species detection |
| Civade et al. (2016) | Spatial representativeness of environmental DNA metabarcoding signal for fish biodiversity assessment in a natural freshwater system | river and lake | Freshwater fish | fish community | France | fish diversity assessment via eDNA metabarcoding |
| Davison et al. (2016) | Laboratory and field validation of a simple method for detecting four species of non-native freshwater fish using eDNA | experimental tanks and ponds | Pumpkinseed, Sunbleak, Fathead Minnow and Topmouth Gudgeon | invasive | UK | invasive species detection |
| Eichmiller et al. (2016) | Optimizing techniques to capture and extract environmental DNA for detection and quantification of fish | experimental tanks | Common Carp | invasive | Minnesota, USA | method optimization of eDNA capture and extraction |
| Erickson et al. (2016) | Detecting the movement and spawning activity of bigheaded carps with environmental DNA | river | Bighead Carp | invasive | Indiana, USA | abundance estimation |
| Eva et al. (2016) | Trails of river monsters: Detecting critically endangered Mekong giant catfish *Pangasianodon gigas* using environmental DNA | artificial ponds and nature river | Mekong Giant Catfish | endangered | Thailand | endangered species detection |
| Furlan & Gleeson (2016) | Environmental DNA detection of redfin perch, *Perca fluviatilis* | streams | Redfin Perch | invasive | Australia | invasive species detection |
| Gingera et al. (2016) | Detection and identification of lampreys in Great Lakes streams using environmental DNA | experimental tanks and natural river | Lampreys | invasive | the Great Lakes, USA | invasive species detection |
| Hanfling et al. (2016) | Environmental DNA metabarcoding of lake fish communities reflects long-term data from established survey methods | lakes | Freshwater fish | fish community | UK | fish diversity assessment and quantification via eDNA metabarcoding |
| Keskin et al. (2016) | Detection of rare and invasive freshwater fish species using eDNA pyrosequencing: Lake Iznik ichthyofauna revised | lake | Freshwater fish | fish community | Bursa, Turkey | fish diversity assessment via eDNA metabarcoding |
| Lacoursiere-Roussel et al. (2016a) | Quantifying relative fish abundance with eDNA: a promising tool for fisheries management | lakes | Lake Trout | common | Canada | abundance and biomass estimation |
| Lacoursiere-Roussel et al. (2016b) | Estimating fish abundance and biomass from eDNA concentrations: variability among capture methods and environmental conditions | experimental aquariums | Brook Charr | common | Canada | abundance and biomass estimation |
| McKelvey et al. (2016) | Sampling large geographic areas for rare species using environmental DNA: A study of bull trout *Salvelinus confluentus* occupancy in western Montana | streams | Bull Trout | threatened | Montana, USA | threatened species detection; eDNA and electrofishing comparison |
| Minamoto et al. (2016) | Techniques for the practical collection of environmental DNA: filter selection, preservation, and extraction | experimental aquariums, natural pond and lake | Common Carp | common | Japan | method optimization of eDNA capture, preservation and extraction |
| Miya et al. (2016) | Use of a filter cartridge for filtration of water samples and extraction of environmental DNA | protocol |  |  | Japan | method optimization of eDNA filtration, preservation and extraction |
| Olds et al. (2016) | Estimating species richness using environmental DNA | stream | Freshwater fish | fish community | Indiana, USA | fish diversity assessment via eDNA metabarcoding |
| Pfleger et al. (2016) | Saving the doomed: Using eDNA to aid in detection of rare sturgeon for conservation (Acipenseridae) | rivers | Rare Sturgeon | endangered | Alabama, USA | endangered species detection |
| Piggott (2016) | Evaluating the effects of laboratory protocols on eDNA detection probability for an endangered freshwater fish | experimental dam | Macquarie Perch | endangered | Australia | endangered species detection |
| Port et al. (2016) | Assessing vertebrate biodiversity in a kelp forest ecosystem using environmental DNA | sea | Marine fish | fish community | Montana, USA | fish diversity assessment via eDNA metabarcoding |
| Robson et al. (2016) | Fine-tuning for the tropics: application of eDNA technology for invasive fish detection in tropical freshwater ecosystems | experimental tanks | Mozambique Tilapia | invasive | Australia | invasive species detection |
| Sassoubre et al. (2016) | Quantification of environmental DNA (eDNA) shedding and decay rates for three marine fish | experimental mesocosms | Northern Anchovy, Pacific Sardine and Pacific Chub Mackerel | common | USA | eDNA production and persistence : eDNA shedding and decay rates quantification |
| Schmelzle & Kinziger (2016) | Using occupancy modelling to compare environmental DNA to traditional field methods for regional-scale monitoring of an endangered aquatic species | lagoon and sea | Tidewater Goby | endangered | California, USA | endangered species detection; eDNA and traditional field methods comparison |
| Shaw et al. (2016) | Comparison of environmental DNA metabarcoding and conventional fish survey methods in a river system | river and lake | Freshwater fish | fish community | Australia | fish diversity assessment via eDNA metabarcoding; eDNA and conventional survey methods comparison |
| Sigsgaard et al. (2016) | Population characteristics of a large whale shark aggregation inferred from seawater environmental DNA | sea | Whale Shark | endangered | Qatar | population genetics analysis via eDNA |
| Simmons et al. (2016) | Active and passive environmental DNA surveillance of aquatic invasive species | rivers | Bighead Carp, Silver Carp and Northern Snakehead | invasive | Ohio, USA | non-target invasive species detection via eDNA metabarcoding |
| Simpfendorfer et al. (2016) | Environmental DNA detects critically endangered largetooth sawfish in the wild | experimental mesocosms; natural rivers, ponds and lagoons | Largetooth Sawfish | endangered | Australia | endangered species detection |
| Thomsen et al. (2016) | Environmental DNA from seawater samples correlate with trawl catches of subarctic, deepwater fishes | sea | Marine fish | fish community | Greenland, Denmark | fish diversity assessment and quantification via eDNA metabarcoding; seawater eDNA and trawling comparison |
| Tucker et al. (2016) | A sensitive environmental DNA (eDNA) assay leads to new insights on Ruffe (*Gymnocephalus cernua*) spread in North America | lakes, rivers and sea | Ruffe | invasive | the Great Lakes, USA | invasive species detection |
| Uchii et al. (2016) | A novel environmental DNA approach to quantify the cryptic invasion of non-native genotypes | experimental aquariums; natural ponds | non-native genotypes of Common Carp | invasive | Japan | invasive species detection |
| Valentini et al. (2016) | Next-generation monitoring of aquatic biodiversity using environmental DNA metabarcoding | ponds, lakes, streams and rivers | Freshwater fish | fish community | France | fish diversity assessment via eDNA metabarcoding; eDNA and conventional survey methods comparison |
| Vences et al. (2016) | Freshwater vertebrate metabarcoding on Illumina platforms using double-indexed primers of the mitochondrial 16S rRNA gene | streams and ponds | Freshwater fishes and amphibians | fish community | Germany | fish diversity assessment via eDNA metabarcoding |
| Wilcox et al. (2016) | Understanding environmental DNA detection probabilities: A case study using a stream-dwelling char *Salvelinus fontinalis* | streams; experimental mesocosms | Brook Trout | invasive | Colorado, USA | method optimization of eDNA detection; eDNA and traditional backpack electrofishing comparison |
| Wilson et al. (2016) | Recognizing false positives: synthetic oligonucleotide controls for environmental DNA surveillance | in silico | Bighead, Silver and Grass Carps | invasive | Canada | method optimization: recognizing false positives |
| Yamamoto et al. (2016) | Environmental DNA as a 'Snapshot' of Fish Distribution: A Case Study of Japanese Jack Mackerel in Maizuru Bay, Sea of Japan | sea | Japanese Jack Mackerel | common | Japan | biomass estimation |
| Yamanaka & Minamoto (2016) | The use of environmental DNA of fishes as an efficient method of determining habitat connectivity | river | Temperate Seabass, Flathead Grey Mullet and Ayu | common | Japan | fish seasonal migration monitoring |
| Yamanaka et al. (2016) | On-site filtration of water samples for environmental DNA analysis to avoid DNA degradation during transportation | pond | Largemouth Bass and Bluegill | invasive | Japan | method optimization: protocol of on-site filtration for water samples |
| Andruszkiewicz et al. (2017a) | Persistence of marine fish environmental DNA and the influence of sunlight | experimental mesocosms; sea | Marine fish | fish community | California, USA | eDNA persistence; fish diversity assessment via eDNA metabarcoding |
| Andruszkiewicz et al. (2017b) | Biomonitoring of marine vertebrates in Monterey Bay using eDNA metabarcoding | sea | Marine fish | fish community | California, USA | fish diversity assessment via eDNA metabarcoding |
| Bakker et al. (2017) | Environmental DNA reveals tropical shark diversity in contrasting levels of anthropogenic impact | sea | Marine fish | fish community | Jamaica | fish diversity assessment via eDNA metabarcoding |
| Balasingham et al. (2017) | Residual eDNA detection sensitivity assessed by quantitative real-time PCR in a river ecosystem | river | Atlantic Salmon | common | Canada | eDNA persistence: residual eDNA detection sensitivity |
| Carim et al. (2017) | A noninvasive tool to assess the distribution of Pacific Lamprey (*Entosphenus tridentatus*) in the Columbia River Basin | river | Pacific Lamprey | threatened | Columbia, USA | threatened species detection |
| Davison et al. (2017) | Application of environmental DNA analysis to inform invasive fish eradication operations | ponds | Topmouth Gudgeon | invasive | UK | invasive species detection |
| Deiner et al. (2017) | Long-range PCR allows sequencing of mitochondrial genomes from environmental DNA | experimental mesocosms; natural streams | Freshwater fish | fish community | Indiana, USA | technology advance: mitochondrial genomes sequencing via eDNA |
| DiBattista et al. (2017) | Assessing the utility of eDNA as a tool to survey reef-fish communities in the Red Sea | sea | Marine fish | fish community | Saudi Arabia | fish diversity assessment via eDNA metabarcoding |
| Doi et al. (2017a) | Water sampling for environmental DNA surveys by using an unmanned aerial vehicle | dam reservoir | Bluegill Sunfish and Largemouth Bass | invasive | Japan | method optimization of eDNA capture |
| Doi et al. (2017b) | Environmental DNA analysis for estimating the abundance and biomass of stream fish | river | *Plecoglossus altivelis* | common | Japan | abundance and biomass estimation |
| Doi et al. (2017c) | Isopropanol precipitation method for collecting fish environmental DNA | experimental mesocosms; natural ponds | Common Carp | common | Japan | method optimization of eDNA capture |
| Erickson et al. (2017) | Seasonal trends in eDNA detection and occupancy of bigheaded carps | river | Silver and Bighead Carps | invasive | Indiana, USA | invasive species detection and quantification |
| Evans et al. (2017a) | Fish community assessment with eDNA metabarcoding: effects of sampling design and bioinformatic filtering | pond | Freshwater fish | fish community | Michigan, USA | fish diversity assessment via eDNA metabarcoding |
| Evans et al. (2017b) | Comparative cost and effort of fish distribution detection via environmental DNA analysis and electrofishing | stream | Brook Trout | invasive | Wisconsin, USA | invasive species detection; eDNA and traditional electrofishing comparison |
| Furlan & Gleeson (2017) | Improving reliability in environmental DNA detection surveys through enhanced quality control | stream | European Carp | common | Australia | method optimization of eDNA detection with positive control |
| Gargan et al. (2017) | Development of a sensitive detection method to survey pelagic biodiversity using eDNA and quantitative PCR: A case study of devil ray at seamounts | sea | Chilean Devil Ray | common | Atlantic, Azores | transient pelagic marine fish detection |
| Hinlo et al. (2017a) | Environmental DNA monitoring and management of invasive fish: comparison of eDNA and fyke netting | streams | Common Carp, Redfin Perch and Oriental Weather Loach | invasive | Australia | invasive species detection; eDNA and fyke netting comparison |
| Hinlo et al. (2017b) | Methods to maximise recovery of environmental DNA from water samples | experimental aquariums; natural stream | Oriental Weather Loach | invasive | Australia | method optimization of eDNA capture, preservation and extraction |
| Hunter et al. (2017) | Detection limits of quantitative and digital PCR assays and their influence in presence-absence surveys of environmental DNA | ponds | Grass Carp | invasive | Florida, USA | method optimization of eDNA detection |
| Jo et al. (2017) | Rapid degradation of longer DNA fragments enables the improved estimation of distribution and biomass using environmental DNA | experimental aquariums; sea | Japanese Jack Mackerel | common | Japan | eDNA persistence: eDNA degradation |
| Klobucar et al. (2017) | At the forefront: evidence of the applicability of using environmental DNA to quantify the abundance of fish populations in natural lentic waters with additional sampling considerations | lakes | Arctic Char | common | Alaska, USA | abundance estimation |
| Lance et al. (2017) | Experimental observations on the decay of environmental DNA from bighead and silver carps | experimental aquariums | Silver and Bighead Carps | invasive | Mississippi, USA | eDNA persistence: degradation |
| Minamoto et al. (2017) | Nuclear internal transcribed spacer-1 as a sensitive genetic marker for environmental DNA studies in common carp *Cyprinus carpio* | experimental mesocosm and pond; natural lake | Common Carp | common | Japan | method optimization: genetic marker development |
| Perez et al. (2017) | Comparison of American Fisheries Society (AFS) standard fish sampling techniques and environmental DNA for characterizing fish communities in a Large Reservoir | lake | Largemouth Bass and Gizzard Shad | common | Arizona, USA | species-specific detection and quantification; eDNA and electrofishing & gillnetting comparison |
| Piggott (2017) | An environmental DNA assay for detecting Macquarie perch, *Macquaria australasica* | rivers | Macquarie Perch | endangered | Australia | endangered species detection |
| Sakata et al. (2017) | Identifying a breeding habitat of a critically endangered fish, *Acheilognathus typus*, in a natural river in Japan | river | *Acheilognathus typus* | endangered | Japan | endangered species detection |
| Sato et al. (2017) | Usefulness and limitations of sample pooling for environmental DNA metabarcoding of freshwater fish communities | lakes | Freshwater fish | fish community | Japan | fish diversity assessment via eDNA metabarcoding |
| Shogren et al. (2017) | Controls on eDNA movement in streams: Transport, Retention, and Resuspension | streams | Common Carp | invasive | Indiana, USA | eDNA persistence: transport, retention, and resuspension |
| Sigsgaard et al. (2017) | Seawater environmental DNA reflects seasonality of a coastal fish community | sea | Marine fish | fish community | Denmark | fish diversity assessment via eDNA metabarcoding |
| Song et al. (2017) | Making sense of the noise: The effect of hydrology on silver carp eDNA detection in the Chicago area waterway system | rivers | Silver Carp | invasive | Chicago, USA | eDNA ecology: the effect of environment on eDNA detection |
| Stoeckle et al. (2017a) | A systematic approach to evaluate the influence of environmental conditions on eDNA detection success in aquatic ecosystems | experimental aquariums | Round Goby | invasive | Germany | eDNA ecology: the effect of environment on eDNA detection |
| Stoeckle et al. (2017b) | Aquatic environmental DNA detects seasonal fish abundance and habitat preference in an urban estuary | rivers | Freshwater fish | fish community | New York, USA | fish diversity assessment and quantification via eDNA metabarcoding |
| Strobel et al. (2017) | Exploring the use of environmental DNA to determine the species of Salmon Redds | rivers | Chinook and Coho Salmon | commercial | Oregon, USA | commercial species detection |
| Tsuji et al. (2017) | Effects of water pH and proteinase K treatment on the yield of environmental DNA from water samples | experimental aquariums and ponds | Common Carp and Ayu | common | Japan | eDNA ecology: the effect of environment on eDNA capture and extraction |
| Uchii et al. (2017) | Distinct seasonal migration patterns of Japanese native and non-native genotypes of common carp estimated by environmental DNA | lake | two genotypes of Common Carp | invasive | Japan | fish seasonal migration monitoring |
| Ulibarri et al. (2017) | Comparing efficiency of American fisheries society standard snorkeling techniques to environmental DNA sampling techniques | streams | Bluehead Suckers and Zuni Bluehead Suckers | common | Arizona and New Mexico, USA | eDNA and snorkeling comparison |
| Wozney & Wilson (2017) | Quantitative PCR multiplexes for simultaneous multispecies detection of Asian carp eDNA | lakes | Bighead, Silver, Grass and Black Carps | invasive | the Great Lakes, USA | invasive species detection |
| Yamamoto et al. (2017) | Environmental DNA metabarcoding reveals local fish communities in a species-rich coastal sea | sea | Marine fish | fish community | Japan | fish diversity assessment via eDNA metabarcoding |
| Yamanaka et al. (2017) | A simple method for preserving environmental DNA in water samples at ambient temperature by addition of cationic surfactant | experimental aquariums; natural ponds and river | Bluegill Sunfish and Ayu Sweetfish | invasive | Japan | method optimization of eDNA preservation |
| Anderson et al. (2018) | Confirmed observation: a North American Green Sturgeon *Acipenser medirostris* recorded in the Stanislaus River, California | rivers | Green Sturgeon | threatened | California, USA | threatened species detection |
| Atkinson et al. (2018) | A quantitative PCR-based environmental DNA assay for detecting Atlantic salmon (*Salmo salar* L.) | rivers | Atlantic Salmon | invasive | Ireland | invasive species detection |
| Balasingham et al. (2018) | Environmental DNA detection of rare and invasive fish species in two Great Lakes tributaries | rivers | Freshwater fish | fish community | Canada | fish diversity assessment via eDNA metabarcoding |
| Bylemans et al. (2018a) | Does size matter? An experimental evaluation of the relative abundance and decay rates of aquatic environmental DNA | experimental aquariums | Common Goldfish | common | Australia | eDNA persistence |
| Bylemans et al. (2018b) | Toward an ecoregion scale evaluation of eDNA metabarcoding primers: A case study for the freshwater fish biodiversity of the Murray-Darling Basin (Australia) | stream and river | Freshwater fish | fish community | Australia | method optimization: evaluation of eDNA metabarcoding primers |
| Cerco et al. (2018) | A fate and transport model for Asian carp environmental DNA in the Chicago area waterways system | rivers | Silver Carp | invasive | Chicago, USA | eDNA persistence: eDNA concentration and decay quantification |
| Chambert et al. (2018) | An analytical framework for estimating aquatic species density from environmental DNA | experimental mesocosms; natural stream | Common Carp | invasive | Idaho, USA | method optimization of density estimation |
| Clusa & Garcia-Vazquez (2018) | A simple, rapid method for detecting seven common invasive fish species in Europe from environmental DNA | river | Freshwater fish | invasive | Spain | invasive species detection |
| Farley et al. (2018) | Primer designs for identification and environmental DNA (eDNA) detection of Gars | experimental aquariums | Gars (7 species) | threatened | USA | threatened species detection |
| Fernandez et al. (2018) | Environmental DNA for freshwater fish monitoring: insights for conservation within a protected area | streams | Brown Trout; Rainbow Trout | threatened; invasive | Spain | threatened and invasive species detection |
| Fukaya et al. (2018) | Estimating fish population abundance by integrating quantitative data on environmental DNA and hydrodynamic modelling | sea | Japanese Jack Mackerel | common | Japan | fish population abundance estimation |
| Ghosal et al. (2018) | Attracting Common Carp to a bait site with food reveals strong positive relationships between fish density, feeding activity, environmental DNA, and sex pheromone release that could be used in invasive fish management | lakes | Common Carp | invasive | Minnesota, USA | method optimization of eDNA detection |
| Hinlo et al. (2018) | Performance of eDNA assays to detect and quantify an elusive benthic fish in upland streams | stream | Oriental Weather Loach | invasive | Australia | invasive species detection and quantification |
| Kamoroff & Goldberg (2018) | An issue of life or death: using eDNA to detect viable individuals in wilderness restoration | experimental mesocosms; natural stream | Comet Goldfish; Brook Trout; Bull Trout | common; invasive; threatened | California, USA | method optimization of eDNA detection |
| Li et al. (2018a) | The effect of filtration method on the efficiency of environmental DNA capture and quantification via metabarcoding | artificial ponds | Freshwater fish | fish community | UK | method optimization of eDNA capture; quantification for eDNA metabarcoding |
| Li et al. (2018b) | Estimating fish alpha- and beta-diversity along a small stream with environmental DNA metabarcoding | stream | Freshwater fish | fish community | USA | fish diversity assessment via eDNA metabarcoding |
| Maruyama et al. (2018) | Environmental DNA analysis as a non-invasive quantitative tool for reproductive migration of a threatened endemic fish in rivers | river | Three-lips | threatened | Lake Biwa, Japan | threatened species detection; seasonal migration monitoring |
| Matter et al. (2018) | A rapid-assessment method to estimate the distribution of juvenile Chinook Salmon in tributary habitats using eDNA and occupancy estimation | rivers | Chinook Salmon | threatened | Alaska, USA | threatened species detection |
| Mizumoto et al. (2018) | Establishing an environmental DNA method to detect and estimate the biomass of Sakhalin taimen, a critically endangered Asian salmonid | experimental aquariums | Sakhalin Taimen | endangered | Japan | invasive species detection and biomass estimation |
| Nakagawa et al. (2018) | Comparing local- and regional-scale estimations of the diversity of stream fish using eDNA metabarcoding and conventional observation methods | rivers | Freshwater fish | fish community | Lake Biwa, Japan | fish diversity assessment via eDNA metabarcoding; eDNA and existing observational data comparison |
| Nukazawa et al. (2018) | Simulating the advection and degradation of the environmental DNA of Common Carp along a river | river | Common Carp | common | Japan | eDNA persistence: advection and degradation |
| Ostberg et al. (2018) | Distribution and seasonal differences in Pacific Lamprey and *Lampetra* spp eDNA across 18 Puget Sound watersheds | sea | Pacific Lamprey | endangered | Washington, USA | endangered species detection |
| Pont et al. (2018) | Environmental DNA reveals quantitative patterns of fish biodiversity in large rivers despite its downstream transportation | river | Freshwater fish | fish community | France | fish diversity assessment via eDNA metabarcoding; eDNA and historical electrofishing data comparison |
| Robinson et al. (2018) | Monitoring the eradication of the highly invasive topmouth gudgeon (*Pseudorasbora parva*) using a novel eDNA assay | ponds | Topmouth Gudgeon | invasive | UK | invasive species detection |
| Roy et al. (2018) | Development of environmental DNA (eDNA) methods for detecting high-risk freshwater fishes in live trade in Canada | artificial tanks | Freshwater fish | invasive | Canada | invasive species detection |
| Sato et al. (2018) | MitoFish and MiFish pipeline: A mitochondrial genome database of fish with an analysis pipeline for environmental DNA metabarcoding | stream and river | Migratory fish | fish community | Japan | fish mitogenome database building for eDNA metabarcoding |
| Schloesser et al. (2018) | Correlating sea lamprey density with environmental DNA detections in the lab | experimental aquariums | Sea Lamprey | invasive | Wisconsin, USA | invasive species detection and quantification |
| Schultz (2018) | Inference of genetic marker concentrations from field surveys to detect environmental DNA using Bayesian updating | rivers | Bighead and Silver Carps | invasive | Chicago, USA | invasive species detection |
| Sepulveda et al. (2018) | Tradeoffs of a portable, field-based environmental DNA platform for detecting invasive northern pike (*Esox lucius*) in Alaska | lakes | Northern Pike | invasive | Alaska, USA | method optimization: development of field-based eDNA approach |
| Shogren et al. (2018) | Water flow and biofilm cover influence environmental DNA detection in recirculating streams | artificial streams | Rainbow Trout | common | Indiana, USA | eDNA persistence: degradation |
| Tillotson et al. (2018) | Concentrations of environmental DNA (eDNA) reflect spawning salmon abundance at fine spatial and temporal scales | stream | Sockeye Salmon | commercial | Alaska, USA | abundance estimation |
| Ushio et al. (2018) | Quantitative monitoring of multispecies fish environmental DNA using high-throughput sequencing | sea | Marine fish | fish community | Japan | fish diversity assessment and quantification via eDNA metabarcoding |
| Xu et al. (2018) | Monitoring seasonal distribution of an endangered anadromous sturgeon in a large river using environmental DNA | river | Chinese Sturgeon | endangered | China | endangered species detection |
| Antognazza et al. (2019) | Environmental DNA as a non-invasive sampling tool to detect the spawning distribution of European anadromous shads (*Alosa* spp.) | river | European Shads | threatened | UK | threatened species detection: spawning monitoring |
| Bracken et al. (2019) | Identifying spawning sites and other critical habitat in lotic systems using eDNA "snapshots": A case study using the sea lamprey *Petromyzon marinus* L. | rivers | Sea Lamprey | threatened | Ireland | threatened species detection: spawning monitoring |
| Cilleros et al. (2019) | Unlocking biodiversity and conservation studies in high-diversity environments using environmental DNA (eDNA): A test with Guianese freshwater fishes | streams and rivers | Freshwater fish | fish community | France | fish diversity assessment via eDNA metabarcoding |
| Harper et al. (2019) | Development and application of environmental DNA surveillance for the threatened crucian carp (*Carassius carassius*) | ponds | Crucian Carp | threatened | UK | threatened species detection |
| Jeunen et al. (2019) | Environmental DNA (eDNA) metabarcoding reveals strong discrimination among diverse marine habitats connected by water movement | sea | Marine fish | fish community | New Zealand | fish diversity assessment via eDNA metabarcoding |
| Jo et al. (2019) | Effect of water temperature and fish biomass on environmental DNA shedding, degradation, and size distribution | experimental tanks | Japanese Jack Mackerels | common | Janpan | eDNA persistence: shedding, degradation, and size distribution |
| Knudsen et al. (2019) | Species-specific detection and quantification of environmental DNA from marine fishes in the Baltic Sea | sea | Atlantic Herring, Atlantic Cod, European Flounder, European Plaice and Atlantic Mackerel | common | Denmark | species-specific detection and quantification |
| Levi et al. (2019) | Environmental DNA for the enumeration and management of Pacific salmon | streams | Pacific Salmon (Sockeye & Coho) | commercial | Alaska, USA | species-specific detection and quantification |
| Li et al. (2019a) | Development of an eDNA method for monitoring fish communities: ground truthing in diverse lakes with characterised fish faunas | lakes | Freshwater fish | fish community | UK | fish diversity assessment via eDNA metabarcoding |
| Li et al. (2019b) | Limited dispersion and quick degradation of environmental DNA in fish ponds inferred by metabarcoding | artificial ponds | Freshwater fish | fish community | UK | fish diversity assessment via eDNA metabarcoding |
| Minamoto et al. (2019) | Real-time polymerase chain reaction assays for environmental DNA detection of three salmonid fish in Hokkaido, Japan: Application to winter surveys | rivers | Dolly Varden and Whitespotted Char; Rainbow Trout | native; invasive | Japan | species-specific detection and quantification |
| Murakami et al. (2019) | Dispersion and degradation of environmental DNA from caged fish in a marine environment | sea | Striped Jack | common | Janpan | species-specific detection and quantification |
| Nardi et al. (2019) | The expansion of exotic Chinook salmon (*Oncorhynchus tshawytscha*) in the extreme south of Patagonia: an environmental DNA approach | experimental aquariums; nature river | Chinook Salmon | invasive | Argentina | invasive species detection |
| Roy et al. (2019) | A habitat risk assessment and breeding site projection for Slackwater darter (*Etheostoma boschungi*) (Percidae) in Alabama and Tennessee USA | ponds | Slackwater Darter | threatened | Tennessee, USA | threatened species detection and quantification |
| Stat et al. (2019) | Combined use of eDNA metabarcoding and video surveillance for the assessment of fish biodiversity | sea | Marine fish | fish community | Australia | fish diversity assessment via eDNA metabarcoding |
| Strickland & Roberts (2019) | Utility of eDNA and occupancy models for monitoring an endangered fish across diverse riverine habitats | streams | Roanoke Logperch | endangered | Virginia and North Carolina, USA | endangered species detection |
| Sutter & Kinziger (2019) | Rangewide tidewater goby occupancy survey using environmental DNA | sea | Tidewater Gobies | endangered | California, USA | endangered species detection |

^*^ If no related information has been specified in the paper, this had been left blank in the table.
