# Supplementary material for: Standards for Methods Utilizing Environmental DNA for Detection of Fish Species
Source: Genes (Basel). 2020 Mar 11;11(3):296. doi: 10.3390/genes11030296 (PMC7140814; doi:10.3390/genes11030296)
Supplement: Supplementary file 1 [file genes-11-00296-s001.zip › genes-712794-supplementary/Supplementary/Table S2.docx]

| Table S2. Summary of sampling volume and depth, eDNA extraction, genetic markers, amplification and sequencing used to detect fish eDNA in water bodies. | | | | | | | | | |
| --- | --- | --- | --- | --- | --- | --- | --- | --- | --- |
| Reference | Environment | Sampling volume & depth | DNA concentration | Filter type ^a^ | Filters preservation | DNA extraction ^b^ | Genetic marker | PCR type | Sequencing technology |
| Jerde et al. (2011) | rivers | 2 L: surface | filtration within 24h | GF filters: 1.5 μm | stored at -20 °C | PowerWater kit | D-loop region | PCR | Sanger Sequencing |
| Dejean et al. (2011) | artificial ponds | 15 mL | centrifugation |  |  | QIAamp kit | D-loop region: 98 bp | PCR |  |
| Minamoto et al. (2011) | experimental aquariums; natural river | aquarium 120 mL; river 2 L: surface | centrifugation |  |  | DNeasy kit | Cytb gene: 285 bp | PCR | Cloning and Sanger Sequencing |
| Takahara et al. (2012) | experimental aquariums and ponds; natural lagoon | aquarium 20 mL; pond or lagoon 2 L: surface | centrifugation; filtration | PC filters: 0.8, 3.0, 12.0 μm | stored at -25/-18 °C | DNeasy kit | Cytb gene: 78 bp | qPCR | Sanger Sequencing |
| Thomsen et al. (2012a) | ponds and streams | 15 mL | centrifugation |  |  | DNeasy kit | Cytb genes: 70 bp | qPCR |  |
| Thomsen et al. (2012b) | sea | 50 mL: 1.5-6 m | filtration | nylon filters: 0.45 μm |  | DNeasy kit | Cytb gene | PCR; qPCR | Cloning and Sanger Sequencing; 454 sequencing |
| Jerde et al. (2013) | the Great Lakes basin (streams, rivers and bays) | 2 L: surface | filtration within 24h | GF filters: 1.5 μm |  | PowerWater kit | D-loop region: 191/312 bp | PCR | Sanger Sequencing |
| Takahara et al. (2013) | ponds | 1 L: surface | filtration | CA filters | stored at -25 °C | DNeasy kit | Cytb gene: 100 bp | qPCR | Sanger Sequencing |
| Wilcox et al. (2013) | streams | 6 L | filtration on-site | GF filters: 1.5 μm | stored on ice to lab | PowerWater kit | Cytb gene | qPCR | Sanger Sequencing |
| Barnes et al. (2014) | experimental mesocosms | 2 L or 250 mL | filtration | Isopore membrane filters: 1.2 μm | stored at -20 °C | CTAB extraction (Coyne et al., 2005) | specific primers: 146 bp | qPCR | Sanger Sequencing |
| Diaz-Ferguson et al. (2014) | experimental aquariums; natural river | 1 L | filtration | CN filters: 0.45 μm | dried at 56 °C for 10 min | Rapid Water kit | COI gene: 64/240 bp | PCR; qPCR |  |
| Kelly et al. (2014) | artificial aquarium | 1 or 15 L | filtration | Durapore membrane filters: 0.22 μm | stored at -80 °C | DNeasy kit | 12S rRNA gene: 106 bp | PCR | Next-Generation DNA Sequencing |
| Keskin (2014) | rivers | 2 L | filtration | Sterivex-GP unit: 0.22 μm |  | PowerWater Kit | COI gene: 150 bp | PCR | Sanger Sequencing |
| MacDonald et al. (2014) | river |  |  |  |  |  | 12S rRNA gene: 205 bp | PCR |  |
| Mahon et al. (2014) | artificial tanks | 2 L | filtration within 24h | GF filters: 1.5 μm |  | PowerWater Kit | COI gene | PCR | Next-Generation DNA Sequencing |
| Turner et al. (2014) | pond and lake | 12 L: ≤1 m | filtration; centrifugation | nylon filters: 0.2, 60, 100, 180 μm | stored at -20 °C | CTAB extraction | specific primers | qPCR |  |
| Wilson et al. (2014) | lakes and rivers | 2 L | filtration within 24h | GF filters: 1.2 μm | stored at -20 °C | Rapid Water kit | COI gene | PCR; qPCR |  |
| Wittmann et al. (2014) | the Great Lakes basin (rivers and lakes) |  |  |  |  |  | see Mahon et al. (2013) |  |  |
| Amberg et al. (2015) | the Great Lakes basin (rivers and lakes) | 2 L | filtration | GF filters: 1.5 μm | stored at -80 °C | DNeasy kit; PowerWater kit | D-loop region | PCR; qPCR |  |
| Doi et al. (2015a) | ponds | 1 L: surface | filtration | CA filters: 3.0 μm |  | DNeasy kit | Cytb gene: 100 bp | qPCR; ddPCR |  |
| Doi et al. (2015b) | experimental mesocosms | 15 mL: surface | centrifugation |  |  | DNeasy kit | Cytb gene | qPCR; ddPCR |  |
| Evans et al. (2015) | experimental mesocosms | 250 mL | filtration on-site | PC filters: 1.2 μm | stored at -20 °C | CTAB extraction | Cytb, 12S rRNA & 16S rRNA genes | PCR | Next-Generation DNA Sequencing |
| Furlan et al. (2015) | streams | 2 L | filtration | GF filters: 1.2 μm | stored at -20 °C | PowerWater kit | 12S rRNA gene | qPCR |  |
| Jane et al. (2015) | streams | 6 L | filtration on-site | GF filters: 1.5 μm | stored at -20 °C | PowerWater kit | Cytb gene | qPCR |  |
| Janosik et al. (2015) | streams | 2 L: surface | filtration within 24h | GF filters: 1.5 μm | stored at -20 °C | PowerWater kit | Cytb gene | PCR | Sanger Sequencing |
| Klymus et al. (2015) | experimental aquariums | 15 mL: 10-15 cm above bottom | centrifugation |  |  | PCI extraction | D-loop region | qPCR |  |
| Laramie et al. (2015) | rivers | 1 L: surface | filtration on-site | CN filters: 0.45 μm |  | DNeasy kit | COI gene: 90 bp | qPCR | Sanger Sequencing |
| Miya et al. (2015) | experimental aquariums; sea | 2L | filtration | GF filters: 0.7 μm | stored at -20 °C | DNeasy kit | 12S rRNA gene: 163-185 bp | PCR | Next-Generation DNA Sequencing |
| Nathan et al. (2015) | artificial tanks | 2L | filtration within 24h | GF filters: 1.5 μm | stored at -20 °C | PowerWater kit | D-loop region: 200-300 bp; Cytb gene:106 bp; COI gene: 150 bp | PCR |  |
| Renshaw et al. (2015) | experimental mesocosms | 250 mL | ethanol precipitation; filtration | CN: 0.45, 0.8 μm; PES: 0.8 μm; PCTE: 1 μm; GF: 1.5 μm | stored at -20 °C | PCI extraction; DNeasy kit; PowerWater kit | Cytb gene: 100 bp | qPCR |  |
| Schultz et al. (2015) | rivers | 2 L: surface | filtration | GF filters: 1.5 μm | stored on dry ice to lab | PowerWater kit | D-loop region | PCR | Sanger Sequencing |
| Sigsgaard et al. (2015) | ponds and streams | 50 mL: surface | centrifugation |  |  | DNeasy kit | COI gene | qPCR | Sanger Sequencing |
| Takahara et al. (2015) | ponds | 1 L | filtration | CA filters: 3.0 μm | stored at -25 °C | DNeasy kit | Cytb gene | qPCR |  |
| Turner et al. (2015) | experimental ponds; natural rivers | 15 mL: surface | centrifugation |  |  | CTAB extraction | D-loop region: 100 bp | qPCR |  |
| Wilcox et al. (2015) | stream | 500 mL | filtration | nylon filters: 60 μm; PCTE filters: 0.4, 1.2, 10 μm | stored at -20 °C | DNeasy kit | Cytb gene | qPCR |  |
| Adrian-Kalchhauser et al. (2016) | ponds and rivers | 500 mL: bottom | ethanol precipitation; filtration | GF filters: 1.5 μm |  | DNeasy kit | Cytb gene | PCR |  |
| Baldigo et al. (2016) | streams | 6 L | filtration | GF filters: 1.5 μm |  | PowerWater kit | Cytb gene | qPCR |  |
| Banks et al. (2016) | streams | 2L | filtration | GF filters: 1.6 μm | stored at -20 °C | PowerWater kit | D-loop region: 148 bp | PCR | Sanger Sequencing |
| Bergman et al. (2016) | river | 2L: 6 inches | filtration on-site | GP sterile filter unit: 0.22 μm | stored at -20 °C | PowerWater kit | COI gene | qPCR |  |
| Boothroyd et al. (2016) | lakes | 1 L | filtration within 24h | GF filters: 1.0, 1.2 μm | stored at -80 °C | PowerWater kit | COI gene: 61 bp | qPCR | Sanger Sequencing |
| Bylemans et al. (2016) | streams | 8*2 L: surface | filtration | GF filters: 1.2 μm | stored at -20 °C | PowerWater kit | 12S rRNA gene | qPCR |  |
| Cannon et al. (2016) | river | 50 mL: surface | centrifugation |  |  | DNeasy kit | Cytb gene: 90 bp | PCR | Next-Generation DNA Sequencing |
| Carim et al. (2016a) | streams and rivers | 5 L |  |  |  | DNeasy kit | COI gene | qPCR |  |
| Carim et al. (2016b) | streams | 5 L |  |  |  | DNeasy kit | Cytb gene | qPCR |  |
| Civade et al. (2016) | river and lake | 45 L | filtration on-site | filtration capsules: 1 μm | filled with a preservation buffer then stored at 4 °C | DNeasy kit | 12S rRNA gene | PCR | Next-Generation DNA Sequencing |
| Davison et al. (2016) | experimental tanks and ponds | tank 1L; pond 125 mL | filtration | PC filters: 0.4 μm | stored at -80 °C | PowerWater kit | COI gene | qPCR |  |
| Eichmiller et al. (2016) | experimental tanks | 15/50/300 mL | ethanol precipitation; centrifugation; filtration | PCTE filters: 0.2, 0.6, 1.0 μm; GF filters: 1.5, 5.0 μm |  | PowerSoil kit; PowerWater kit; MP kit; DNeasy kit; QIAamp Kit | Cytb gene | qPCR |  |
| Erickson et al. (2016) | river | 50 mL: surface | centrifugation |  |  | DNeasy kit | D-loop region | qPCR |  |
| Eva et al. (2016) | artificial ponds and nature river | 10 L: surface, middle and near-bottom | filtration within 24h | filtration capsules: 1 μm | stored at 4 °C | DNeasy kit | Cytb gene | qPCR |  |
| Furlan & Gleeson (2016) | streams | 2 L | filtration | GF filters: 1.2 μm | stored at -20 °C | PowerWater kit | 12S rRNA gene: 92 bp | qPCR |  |
| Gingera et al. (2016) | experimental tanks and natural river | tank 500 mL; river 1-2 L | filtration on-site | GF filters: 1.5 μm | stored at -30 °C | DNeasy kit | COI gene | PCR |  |
| Hanfling et al. (2016) | lakes | 5*400 mL: 2, 10 and 20 m | filtration | CN filters: 0.45 μm |  | PowerWater kit | 12S rRNA: 106 bp; Cytb gene: 460 bp | PCR | Next-Generation DNA Sequencing |
| Keskin et al. (2016) | lake | 1.5 L: 1-5 m | filtration within 48 h | CN filters: 0.22 μm |  | EURx kit | Cytb gene: 130 bp | PCR | Next-Generation DNA Sequencing |
| Lacoursiere-Roussel et al. (2016a) | lakes | 1 L: 0-5 m | filtration within 24 h | GF filters: 1.2 μm | stored at -20 °C | DNeasy kit | COI gene: 66 bp | qPCR |  |
| Lacoursiere-Roussel et al. (2016b) | experimental aquariums | 1 L | filtration within 2 h | MCE: 0.2, 0.45 μm; GF filters: 0.7, 1.2, 3.0 μm | stored at -20 °C | Salt extraction (Aljanabi & Martinez 1997) | Cytb gene: 139 bp | qPCR |  |
| McKelvey et al. (2016) | streams | 5 L | filtration on-site | GF filters: 1.5 μm | stored at -20 °C | DNeasy kit | specific primers | qPCR |  |
| Minamoto et al. (2016) | experimental aquariums, natural pond and lake | 500 mL | ethanol precipitation; filtration | GF filters: 0.7, 1.6, 2.7 μm; PC filters: 0.2, 0.8, 3.0 μm | ethanol preservation at ambient temperatures | phenol treatment or ultrafiltration then DNeasy kit | Cytb gene: 78 bp | qPCR |  |
| Miya et al. (2016) | protocol | ≤4 L or >4 L | filtration | the Filter Cartridge |  | PCI extraction; DNeasy kit |  |  |  |
| Olds et al. (2016) | stream | 250 mL | filtration | PC filters: 1.2 μm | stored at -20 °C | modified PCI extraction (Renshaw et al. 2015) | Cytb, 12S rRNA & 16S rRNA genes | PCR | Next-Generation DNA Sequencing |
| Pfleger et al. (2016) | rivers | 1 L | filtration within 24 h | GF filters: 1.5 μm | stored at -20 °C | PowerWater kit | Cytb gene: 150 bp | PCR | Sanger Sequencing |
| Piggott (2016) | experimental dam | 250 mL: surface | ethanol precipitation; filtration | CN filters: 0.45 μm | stored at -20 °C | DNeasy kit; PCI extraction | 12S rRNA gene: 78 bp | PCR; qRCR |  |
| Port et al. (2016) | sea | 3 L: 1 m above bottom | filtration on-site |  | stored at -80 °C | PowerWater kit | 12S rRNA gene: 106 bp | PCR | Next-Generation DNA Sequencing |
| Robson et al. (2016) | experimental tanks | 2 L | filtration | filters: 3, 10, 20 μm | stored at -20 °C | Bioline kit | 16S rRNA gene | qPCR |  |
| Sassoubre et al. (2016) | experimental mesocosms | 500 mL | filtration | PCTE filters: 0.2 μm | stored at -20 °C | DNeasy kit | D-loop region; COI gene | qPCR |  |
| Schmelzle & Kinziger (2016) | lagoon and sea | 2 L: bottom and up | filtration | PCTE filters: 3.0 μm | stored at -20 °C | DNeasy kit | Cytb gene | qPCR |  |
| Shaw et al. (2016) | river and lake | 1L: 10-30 cm | filtration | CN filters: 0.45 μm | stored at -20 °C | PowerSoil kit | 12S or 16S rRNA genes | PCR | Next-Generation DNA Sequencing |
| Sigsgaard et al. (2016) | sea | 1.5 L: surface | filtration | Sterivex-GP filters: 0.22 μm |  | DNeasy kit | two polymorphic regions of D-loop region | qPCR; PCR | Sanger Sequencing; high-throughput DNA sequencing |
| Simmons et al. (2016) | rivers | 2 L: surface | filtration within 24 h | GF filters: 1.5 μm | stored at -20 °C | PowerWater kit | D-loop region, COI & 16S rRNA genes; 16S rRNA gene | PCR; ddPCR | Next-Generation DNA Sequencing |
| Simpfendorfer et al. (2016) | experimental mesocosms; natural rivers, ponds and lagoons | 2 L | filtration | nylon filters: 20 μm | stored at -20 °C | Bioline kit | COI gene: 145 bp | PCR | Sanger Sequencing |
| Thomsen et al. (2016) | sea | 2 L: 188-918 m | filtration | nylon filters: 0.45 μm |  | DNeasy kit | 12S rRNA gene: 100 bp | PCR | Next-Generation DNA Sequencing |
| Tucker et al. (2016) | lakes, rivers and sea | 2 L: surface | filtration within 24 h | GF filters: 1.5 μm | stored at -20 °C | PowerWater kit | COI gene | qPCR |  |
| Uchii et al. (2016) | experimental aquariums; natural ponds | tank 500 mL; pond 1 L | filtration | GF filters: 0.7 μm | stored at -20 °C | DNeasy kit | D-loop region | qPCR |  |
| Valentini et al. (2016) | ponds, lakes, streams and rivers | 300 mL; 100 L | centrifugation; filtration on-site | filtration capsules: 1 μm | stored at -20 °C | DNeasy kit | 12S rRNA gene | PCR | Next-Generation DNA Sequencing |
| Vences et al. (2016) | streams and ponds | 1-2 L | filtration | CN filters: 0.45 μm |  | PowerWater kit | 16S rRNA gene: 250 bp | PCR | Next-Generation DNA Sequencing |
| Wilcox et al. (2016) | streams; experimental mesocosms | 6 L | filtration on-site | GF filters: 1.5 μm | stored at -20 °C | DNeasy kit | Cytb gene | qPCR |  |
| Wilson et al. (2016) | in silico |  |  |  |  |  | COI gene | qPCR |  |
| Yamamoto et al. (2016) | sea | 3 L: surface and 1.5 m above bottom | filtration | GF filters: 0.7 μm | stored at -20 °C | DNeasy kit | Cytb gene | qPCR |  |
| Yamanaka & Minamoto (2016) | river | 2 L | filtration | GF filters: 0.7 μm | stored at -20 °C | DNeasy kit | Cytb gene | qPCR |  |
| Yamanaka et al. (2016) | pond | 12L | filtration on-site | GF filters: 0.7 μm | stored at -20 °C | DNeasy kit | Cytb gene | qPCR |  |
| Andruszkiewicz et al. (2017a) | experimental mesocosms; sea | 500 mL | filtration | PCTE filters: 0.22 μm | stored at -80 °C | DNeasy kit | COI gene; 12S rRNA gene | qPCR; PCR | Next-Generation DNA Sequencing |
| Andruszkiewicz et al. (2017b) | sea | 1 L: 0, 20 and 40 m | filtration | PCTE filters: 0.22 μm | stored at -80 °C | DNeasy kit | 12S rRNA gene | PCR | Next-Generation DNA Sequencing |
| Bakker et al. (2017) | sea | 4 L | filtration | MCE filters: 0.45 μm | stored at -20 °C | PowerSoil kit | COI gene | PCR | Next-Generation DNA Sequencing |
| Balasingham et al. (2017) | river | 500 mL: surface | filtration | GF filters: 1.2 μm | stored at -20 °C | CTAB extraction | D-loop region: 230 bp | qPCR |  |
| Carim et al. (2017) | river | 5 L | filtration |  | stored at -20 °C | DNeasy kit | COI gene: 126 bp | qPCR |  |
| Davison et al. (2017) | ponds | 1 L | filtration within 24 h | PC filters: 0.4 μm | stored at -20 °C | PowerWater kit | COI gene | PCR |  |
| Deiner et al. (2017) | experimental mesocosms; natural streams | 250 mL | filtration | PC filters: 1.2 μm | stored at -20 °C | CTAB extraction | 16S rRNA gene: 16-17 kb | long-range PCR | Next-Generation DNA Sequencing |
| DiBattista et al. (2017) | sea | the reef 500 mL | filtration | nylon filters: 0.45 μm | stored at -20 °C | DNeasy kit | 16S rRNA gene: 200 bp | qPCR | Next-Generation DNA Sequencing |
| Doi et al. (2017a) | dam reservoir | 1 L: surface | filtration | GF filters: 0.7 μm |  | DNeasy kit | Cytb gene | qPCR |  |
| Doi et al. (2017b) | river | 1 L: surface | filtration | GF filters: 0.7 μm | stored at -20 °C | DNeasy kit | Cytb gene: 131 bp | qPCR |  |
| Doi et al. (2017c) | experimental mesocosms; natural ponds | 1.5/15/27 mL: surface | isopropanol or ethanol precipitation |  |  | DNeasy kit | Cytb gene | qPCR |  |
| Erickson et al. (2017) | river | 50 mL: surface | centrifugation |  |  | DNeasy kit | ND5 gene | qPCR |  |
| Evans et al. (2017a) | pond | 250 mL: surface | filtration | PC filters: 1.2 μm | stored at -80 °C | modified PCI extraction | Cytb, 12S rRNA & 16S rRNA genes | PCR | Next-Generation DNA Sequencing |
| Evans et al. (2017b) | stream | 2 L | filtration | GF filters: 1.5 μm | stored at -20 °C | PowerWater kit | Cytb gene: 150 bp | qPCR |  |
| Furlan & Gleeson (2017) | stream | 2 L | filtration | GF filters: 1.2 μm | stored at -20 °C | PowerWater kit | 12S rRNA gene: 73 bp; 16S rRNA gene: 87-88 bp | qPCR | Sanger Sequencing |
| Gargan et al. (2017) | sea | 5 L: 5-10 m | filtration | nylon filters: 0.45 μm |  | DNeasy kit | COI gene: 86 bp | qPCR |  |
| Hinlo et al. (2017a) | streams | 2 L: 0 and 0.3 m | filtration | GF filters: 1.2 μm | stored at -20 °C | PowerWater kit | 12S rRNA gene | qPCR | Sanger Sequencing |
| Hinlo et al. (2017b) | experimental aquariums; natural stream | tank 250 mL; stream 20 L | ethanol precipitation; filtration | CN/MCN/PES/PCE/GF filters: 0.8 μm | freeze or ethanol preservation at ambient temperatures | PCI extraction; DNeasy kit; PowerWater kit | 12S rRNA gene | qPCR | Sanger Sequencing |
| Hunter et al. (2017) | ponds | 1 L | filtration |  |  | PCI extraction | specific primers: 61 bp | qPCR; ddPCR |  |
| Jo et al. (2017) | experimental aquariums; sea | 1 L | filtration | GF filters: 0.7 μm | stored at -20 °C | DNeasy kit | Cytb gene: 127/719 bp | qPCR |  |
| Klobucar et al. (2017) | lakes | 5 L: shallow and deep depths | filtration on-site | nylon filters: 10 μm | stored at -80 °C | DNeasy kit | Cytb gene: 145 bp | qPCR |  |
| Lance et al. (2017) | experimental aquariums |  | centrifugation |  |  | CTAB extraction | D-loop region | qPCR |  |
| Minamoto et al. (2017) | experimental mesocosm and pond; natural lake | mesocosm 15 mL; pond 2 L; lake 10 L | ethanol precipitation; filtration | GF filters: 0.7 μm |  | DNeasy kit | COI & ND4 genes; ND3 & ND5 genes | qPCR |  |
| Perez et al. (2017) | lake | 1 L: surface | centrifugation |  |  | IBI kit | Cytb gene: 127 bp | qRCR |  |
| Piggott (2017) | rivers | 1 L: surface | filtration | CN filters: 0.45 μm | stored at -20 °C | DNeasy kit | Cytb gene | qRCR |  |
| Sakata et al. (2017) | river | 1 L | filtration within 48 h | GF filters: 0.7 μm |  | DNeasy kit | 12S rRNA gene: 163-185 bp | PCR | Next-Generation DNA Sequencing |
| Sato et al. (2017) | lakes | 250 mL/500 mL: 1-2 m | filtration | GF filters: 0.7 μm | stored at -20 °C | DNeasy kit | Cytb gene | qRCR |  |
| Shogren et al. (2017) | streams | 250 mL | filtration | PC filters: 1.2 μm |  | CTAB extraction | 12S rRNA gene: 60 bp | PCR | Next-Generation DNA Sequencing |
| Sigsgaard et al. (2017) | sea | 500 mL: surface | filtration on-site | PES filters: 0.22 μm | stored at -18 °C | DNeasy kit |  |  |  |
| Song et al. (2017) | rivers |  |  |  |  |  | COI gene: 130 bp | qRCR |  |
| Stoeckle et al. (2017a) | experimental aquariums |  | filtration | GF filters: 0.4 μm | stored at -80 °C | DNeasy kit | 12S rRNA gene | PCR | Next-Generation DNA Sequencing |
| Stoeckle et al. (2017b) | rivers | 500 mL | filtration | nylon filters: 0.45 μm | stored at -20 °C | PowerWater kit | COI gene: 90 bp; Cytb gene: 114bp | qRCR |  |
| Strobel et al. (2017) | rivers | 15 mL: 2-5 cm and 5-10 cm | centrifugation |  |  | DNeasy kit | Cytb gene | qRCR |  |
| Tsuji et al. (2017) | experimental aquariums and ponds | 100/500 mL | filtration | GF filters: 0.7 μm | stored at -20 °C | DNeasy kit | SNPs; Cytb gene | qRCR |  |
| Uchii et al. (2017) | lake | 2 L: surface | filtration | GF filters: 0.7 μm | stored at -20 °C | DNeasy kit | ND2 gene: 246 bp | qRCR |  |
| Ulibarri et al. (2017) | streams | 50 mL | centrifugation |  |  | IBI kit |  | qRCR |  |
| Wozney & Wilson (2017) | lakes |  |  |  |  |  | 12S rRNA gene:163-185 bp | PCR | Next-Generation DNA Sequencing |
| Yamamoto et al. (2017) | sea | 1 L: surface and bottom | filtration | GF filters: 0.7 μm |  | DNeasy kit | Cytb gene | qRCR |  |
| Yamanaka et al. (2017) | experimental aquariums; natural ponds and river | 500 mL: surface | filtration on-site ; filtration within 6 h-10 days | GF filters: 0.7 μm |  | DNeasy kit | COI gene | qRCR |  |
| Anderson et al. (2018) | rivers | 2 L | filtration on-site | Millipore Sterivex GP filters: 0.45 μm | stored at -20 °C | PowerWater kit | COI & Cytb genes | qRCR |  |
| Atkinson et al. (2018) | rivers | 2 L | filtration | GF filters: 1.5 μm | stored at -20 °C | CTAB extraction | COI gene | PCR | Next-Generation DNA Sequencing |
| Balasingham et al. (2018) | rivers | 500 mL: surface | filtration within 24 h | GF filters: 1.2 μm |  | CTAB extraction | COI gene: 96/285/515 bp; ITS gene: 95 bp | qRCR |  |
| Bylemans et al. (2018a) | experimental aquariums | 50 mL | filtration | GF filters: 1.2 μm |  | PowerWater kit |  |  |  |
| Bylemans et al. (2018b) | stream and river |  |  |  |  |  |  |  |  |
| Cerco et al. (2018) | rivers |  |  |  |  |  |  |  |  |
| Chambert et al. (2018) | experimental mesocosms; natural stream |  |  |  |  |  | 16S rRNA gene | PCR |  |
| Clusa & Garcia-Vazquez (2018) | river | 1 L |  |  |  |  | gar-specific primers | PCR | Sanger Sequencing |
| Farley et al. (2018) | experimental aquariums |  |  |  |  |  | COI gene: 61 bp; NADH gene: 102 bp; 16S rRNA gene | qPCR; PCR-RFLP |  |
| Fernandez et al. (2018) | streams | 1.5 L | filtration | Supor-200 Membrane Filter: 0.2 μm | stored at -20 °C | PowerWater kit | Cytb gene | qPCR |  |
| Fukaya et al. (2018) | sea | 1 L: surface, middle and bottom | filtration | GF filters: 0.7 μm | stored at -20 °C | DNeasy kit | Cytb gene | qPCR |  |
| Ghosal et al. (2018) | lakes | 1 L | filtration | GF filters | stored at -80 °C | MP kit | 12S rRNA gene | qPCR |  |
| Hinlo et al. (2018) | stream | 2 L | filtration | GF filters:1.2 μm |  | DNeasy kit | Cytb gene | qPCR |  |
| Kamoroff & Goldberg (2018) | experimental mesocosms; natural stream | tank 50 mL ; stream 250 mL | filtration | PCTE filters: 0.4, 1.2, 5, 10 μm |  | DNeasy kit |  |  |  |
| Li et al. (2018a) | artificial ponds | 1 L: surface | filtration within 24 h | MCE: 0.45, 0.8 or 1.2 μm; Sterivex: 0.45 μm | stored at -20 °C | PowerWater kit | 12S rRNA: 106 bp, Cytb gene: 460 bp | PCR | Next-Generation DNA Sequencing |
| Li et al. (2018b) | stream | 250 mL | filtration (within 6 h) | PC filters: 1.2 μm | stored at -20 °C | PCI extraction | Cytb, 12S & 16S rRNA genes | PCR | Next-Generation DNA Sequencing |
| Maruyama et al. (2018) | river | 500 mL | filtration on-site | GF filters: 0.7 μm |  | DNeasy kit | COI gene: 90 bp | qPCR |  |
| Matter et al. (2018) | rivers | 1 L: surface | filtration | CN filters: 0.45 μm | ethanol preservation at ambient temperatures | PCI extraction |  | qPCR |  |
| Mizumoto et al. (2018) | experimental aquariums |  |  |  |  |  | 12S rRNA gene:163-185 bp | PCR | Next-Generation DNA Sequencing |
| Nakagawa et al. (2018) | rivers | 1 L: surface | filtration on-site | GF filters: 0.7 μm | stored at -20 °C | DNeasy kit | Cytb gene | dPCR |  |
| Nukazawa et al. (2018) | river | 1/2/10 L | filtration | GF filters: 0.7 μm |  | DNeasy kit | Cytb gene | qPCR |  |
| Ostberg et al. (2018) | sea | 1 L: surface | filtration within 24 h | CN filters: 0.45 μm | stored at -20 °C | DNeasy kit |  | PCR | Next-Generation DNA Sequencing |
| Pont et al. (2018) | river | 30 L | filtration on-site | fltration capsule: 0.45 μm |  |  | 16S rRNA gene: 147 bp | PCR; qPCR |  |
| Robinson et al. (2018) | ponds | 15/750 mL | filtration | GF filters: 0.45 μm | stored at -20 °C | DNeasy kit | COI gene | qPCR |  |
| Roy et al. (2018) | artificial tanks | 850 mL | filtration within 24 h | PES filters: 0.2 μm | stored at -80 °C | PowerWater kit |  |  |  |
| Sato et al. (2018) | stream and river |  |  |  |  |  | Cytb gene; ND1 gene | qPCR |  |
| Schloesser et al. (2018) | experimental aquariums | 1 L | filtration on-site | GF filters: 1.5 μm | stored at -80 °C | IBI kit | D-loop gene | qPCR | Sanger Sequencing |
| Schultz (2018) | rivers | 2 L: surface | filtration | GF filters: 1.5 μm | stored at -20 °C | DNeasy kit | COI gene | qPCR |  |
| Sepulveda et al. (2018) | lakes | 500 mL | on-site or lab filtration | MCE filters: 1.0 μm; GF filters: 1.2 μm | stored at -20 °C | Biomeme kit (field-based eDNA platform); DNeasy kit | specific primers: 97/347/697 bp | qPCR |  |
| Shogren et al. (2018) | artificial streams | 250 mL | filtration within 30 min |  |  |  | COIII gene: 70 bp | qPCR |  |
| Tillotson et al. (2018) | stream | 1 L | filtration | CN filters: 0.45 μm | ethanol preservation at ambient temperatures | DNeasy kit | 12S rRNA gene:163-185 bp | PCR; qPCR | Next-Generation DNA Sequencing |
| Ushio et al. (2018) | sea | 1 L | filtration | GF filters: 0.7 μm | stored at -20 °C | DNeasy kit | ND4 gene | Nested PCR | Sanger Sequencing |
| Xu et al. (2018) | river | 2 L: surface | filtration within 8 h | PES filters: 0.45 μm | stored at -20 °C | PowerWater kit | COI gene | qPCR |  |
| Antognazza et al. (2019) | river | 1 L | filtration |  |  |  | COI gene | qPCR |  |
| Bracken et al. (2019) | rivers | 1 L | filtration within 24 h | CN filters: 0.45 μm | stored at -20 °C | Chelex®Chelating resin (Estoup et al. 1996) | 12S rRNA gene | PCR | Next-Generation DNA Sequencing |
| Cilleros et al. (2019) | streams and rivers | 2 L | filtration on-site | sterile filtration capsule | filled with 150 mL preservation buffer | DNeasy kit | Cytb gene: 118 bp | qPCR | Sanger Sequencing |
| Harper et al. (2019) | ponds | 2 L: surface | filtration | CN filters: 0.45 μm | stored at -20 °C | PowerWater kit | 16S rRNA gene | PCR | Next-Generation DNA Sequencing |
| Jeunen et al. (2019) | sea | 2 L: surface | filtration within 30 min | CN filters: 1.2 μm | stored at -20°C | PCI extraction | Cytb gene: 127bp | qPCR |  |
| Jo et al. (2019) | experimental tanks | 500 mL/1 L | filtration | GF filters: 0.7 μm; PC filters: 0.2, 0.4, 0.8, 3, 10 μm | stored at -20°C | DNeasy kit | Cytb gene; ND4 gene | qPCR |  |
| Knudsen et al. (2019) | sea | 5 L: 37.8-93.8 m | filtration | PES: 0.22 μm | stored at -20°C | DNeasy kit | COI gene | qPCR |  |
| Levi et al. (2019) | streams | 1 L | filtration | CN filters: 0.45 μm | stored in 100% ethanol at 4 °C | DNeasy kit | 12S rRNA: 106 bp | PCR | Next-Generation DNA Sequencing |
| Li et al. (2019a) | lakes | 2 L: surface | filtration within 24 h | MCE filters: 0.45 μm | stored at -20 °C | PowerWater kit | 12S rRNA: 106 bp | PCR | Next-Generation DNA Sequencing |
| Li et al. (2019b) | artificial ponds | 2 L: surface | filtration within 24 h | MCE filters: 0.8 μm | stored at -20 °C | PowerWater kit | D-loop gene: 129 bp | qPCR |  |
| Minamoto et al. (2019) | rivers | 900 mL | filtration | GF filters: 0.7 μm | stored at -20°C | DNeasy kit | Cytb gene: 130 bp | qPCR |  |
| Murakami et al. (2019) | sea | 2L: surface | filtration | GF filters: 0.7 μm | stored at -20°C | DNeasy kit | COI gene; D-loop region | qPCR |  |
| Nardi et al. (2019) | experimental aquariums; nature river | 1 L | filtration | CN filters: 0.45 μm | stored at -80°C | DNeasy kit | Cytb gene | PCR |  |
| Roy et al. (2019) | ponds | 15 mL: surface | centrifugation |  |  | DNeasy kit |  |  |  |
| Stat et al. (2019) | sea | 500 mL: surface | filtration | PES filters: 0.45 μm | stored at -20°C | DNeasy kit | Cytb gene | qPCR |  |
| Strickland & Roberts (2019) | streams | 250 mL: surface | filtration on-site | PCTE filters: 1.0 μm |  | CTAB extraction | Cytb gene: 119 bp | qPCR |  |
| Sutter & Kinziger (2019) | sea | 2 L: surface | filtration on-site | PCTE filters: 3.0 μm | stored at -20°C | DNeasy kit |  |  |  |

^*^ If no related information has been specified in the paper, this had been left blank in the table.

^a^ The filter material abbreviation represents that cellulose acetate (CA); cellulose nitrate (CN); mixed cellulose acetate and nitrate (MCE); mixed cellulose nitrate (MCN); glass fiber (GF); polycarbonate (PC); polycarbonate track-etched (PCTE) or polyethersulfone (PES).

^b^ The detailed name of commercial kits is Bioline kit: Isolate II Genomic DNA Kit (Bioline); Biomeme kit: Biomeme Field Test Kit; CTAB extraction: Hexadecyltrimethy ammonium bromide extraction; DNeasy kit: DNeasy Blood and Tissue Kit (Qiagen); EURx kit: GeneMATRIX Bio-Trace DNA Purification Kit (EURx); IBI kit: gMAX Mini Genomic DNA Kit (IBI); MP kit: FastDNA Spin Kit (MP); PCI extraction: PhenolChloroform-Isoamyl alcohol extraction; PowerSoil kit: PowerSoil DNA Isolation kit (MoBio); PowerWater kit: PowerWater DNA Isolation kit (MoBio); QIAamp kit: QIAamp DNA Stool Mini Kit (Qiagen) or Rapid Water kit: Rapid Water DNA isolation kit (MoBio).
